# Unraveling the Anticancer Effect of Curcumin and Resveratrol

**DOI:** 10.3390/nu8110628

**Published:** 2016-11-10

**Authors:** Aline Renata Pavan, Gabriel Dalio Bernardes da Silva, Daniela Hartmann Jornada, Diego Eidy Chiba, Guilherme Felipe dos Santos Fernandes, Chung Man Chin, Jean Leandro dos Santos

**Affiliations:** School of Pharmaceutical Sciences, UNESP–Univ Estadual Paulista, Araraquara 14800903, Brazil; alinerenatapavan2004@yahoo.com.br (A.R.P.); gabriel.dalio@hotmail.com (G.D.B.d.S.); daniela.hj@hotmail.com (D.H.J.); chiba.diego@outlook.com (D.E.C.); guilhermefelipe@outlook.com (G.F.d.S.F.); chungmc@fcfar.unesp.br (C.M.C.)

**Keywords:** cancer, resveratrol, curcumin, polyphenols, anticancer

## Abstract

Resveratrol and curcumin are natural products with important therapeutic properties useful to treat several human diseases, including cancer. In the last years, the number of studies describing the effect of both polyphenols against cancer has increased; however, the mechanism of action in all of those cases is not completely comprehended. The unspecific effect and the ability to interfere in assays by both polyphenols make this challenge even more difficult. Herein, we analyzed the anticancer activity of resveratrol and curcumin reported in the literature in the last 11 years, in order to unravel the molecular mechanism of action of both compounds. Molecular targets and cellular pathways will be described. Furthermore, we also discussed the ability of these natural products act as chemopreventive and its use in association with other anticancer drugs.

## 1. Introduction

Over the last years, the number of searchers involving polyphenols has increased meaningly. The major reason for that includes the presence of these compounds in our diet contributing to prevention of several diseases. In addition, potent antioxidant properties of polyphenols reduce oxidative stress-associated with some diseases, including cancer. It has been described that polyphenols inhibit carcinogenesis and induce tumor cell death [1].

Among the polyphenols, the interest in two of them has increased in the last years. Papers describing curcumin and/or resveratrol are present in almost fifteen thousand of publications in the last ten years. Both polyphenols have been described as promising anticancer compounds; however, the mode of action for them are still unclear and not fully comprehended [2].

Curcumin (diferuloylmethane) is an active ingredient of the perennial herb *Curcuma longa*, also known as turmeric. The yellow color of this polyphenol is chemically related to its major fraction, which contains curcuminoids [3]. Curcumin has been used for a long time in countries such as China and India as traditional medicines. This ancient remedy has brought the attention of scientific community for a wide range of beneficial properties including anti-inflammatory, antioxidant and chemopreventive [4,5].

By the other hand, resveratrol (*trans-*3,5,4′-trihydroxystilbene) is a stilbene phytoalexin synthetized by a variety of plants, specially vine in response to fungi infections and ultraviolet radiation [6]. This compound is found at high concentration in grapes and red wine, which antioxidant effect is well established in several different assays. Resveratrol has been investigated as potential compound for the treatment of several diseases, regulation of immune system and chemoprevention [7,8].

In clinical studies, the common issue regarding both compounds is the reduced aqueous solubility and low bioavailability [3,9,10,11]. In order to overcome these limitations, studies have been conducted using several strategies. For curcumin, for example, these strategies include: (a) complexation with metal ions, such as Zn^2+^, Cu^2+^, Se^2+^ and Mg^2+^ [12]; (b) co-administration with piperine, which inhibits the phase II metabolism of curcumin and increases its bioavailability [13,14]; (c) Pharmaceutical technologies such as micelles formation and nanoencapsulation were used to increase the bioavailability of curcumin [15,16,17,18,19,20,21,22,23]. Resveratrol has been extensively studied aiming to enhance its aqueous solubility and bioavailability and a number of techniques were used to achieve this goal [24], including: (a) nanoencapsulation [25,26,27,28]; (b) prodrug approach [29]; and (c) co-administration with piperine [30]. These polyphenols have exhibited very low or not-observed toxic effects at daily intake of 0–3 mg·kg^−1^ body weight for curcumin [3] and 0.073 mg–5 g for resveratrol [31]. However, in humans at high doses either curcumin and resveratrol can cause side effects such as diarrhea, skin rash, and headaches [3,31,32,33,34].

Another concern about these both polyphenols is the ability to perturb membranes and alter protein function, that leads to false-results in a series of assays described in the literature [35,36,37]. Therefore, this review article proposes to investigate the real mechanisms involved in the anticancer effect of resveratrol and curcumin in order to clarify the mode of action of both compounds as anticancer drugs useful for prevention and treatment.

## 2. Cell Proliferation

**Figure 1:**
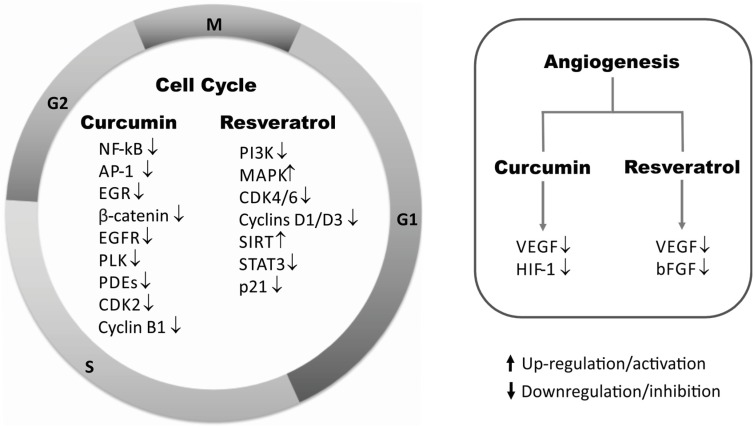
Effects of curcumin and resveratrol in cellular proliferation and angiogenesis.

The antiproliferative effects of curcumin and resveratrol are associated with the modulation of transcription factors, protein kinases, cell cycle regulatory proteins, and inhibition of angiogenesis [9,10]. Some targets related to its effect are presented as following (Figure 1).

### 2.1. Transcription Factors

#### 2.1.1. NF-κB

Nuclear Factor-kappa B (NF-κB) is a pro-inflammatory transcription factor that regulates the expression of more than 200 genes, which are involved in innate and adaptive immunity, cellular transformation, proliferation, antiapoptosis, angiogenesis, invasion and metastasis [38]. Moreover, NF-κB regulates several pro-inflammatory cytokines including, IL-1, IL-2, IL-6, TNF-α and monocyte chemotactic protein 1 (MCP-1). These cytokines are released in chronic inflammation states associated to various cancers [39,40,41,42].

NF-κB is found in an inactive state in the cytoplasm and its activation occurs through the action of a variety of stimuli, such as, carcinogens, mitogens, chemotherapeutic agents, radiation, hypoxia, protein kinases, and degradation of the NF-κB cytoplasmic inhibitor (I-κB) [43,44,45]. Subsequently its activation, NF-κB translocate to the cell nucleus and binds to the target DNA gene promoter region [46].

Luciferase assay was performed transfecting series of plasmids into PC-3 cells with luciferase reporter gene. The data showed down regulation of NF-κB blocking the development and progression of prostate cancer cells (PC-3) [47].

Curcumin showed a potent antiproliferative effect on melanoma cell lines by NF-κB inhibition. Three melanoma cell lines were treated with curcumin and it has shown a decreasing of NF-κB binding activity through electrophoretic mobility shift assay (EMSA), and an inhibition of cell viability in a dose-dependent manner with IC_50_ ranging from 6.1 µM to 7.7 µM [48].

#### 2.1.2. AP-1

The activating protein-1 (AP-1) transcription factor is related to control an extensive range of cellular processes, including cell proliferation. Dysfunctions in the AP-1 transcription factor levels are associated to the growth and progression of many types of cancer [49]. AP-1 showed to be required for binding in the involucrin (hINV), which is a marker of keratinocyte differentiation [50].

Using a High-Throughput Cell-Based Assay, it was identified potentials AP-1 inhibitors. In this assay, curcumin has shown inhibiting AP-1 in the dose-dependent manner with IC_50_ values of 100 μM [51].

In a different study, using fluorescent cell-staining assay it was shown that curcumin also suppress the in vitro growth of PC-3 cells. By a luciferase assay, it was determined the intracellular signal pathway via inhibition of androgen-induced AP-1 activity in prostate cancer cells (PC-3). Flow cytometry data indicated that curcumin arrested 57.29% of PC-3 cells in G2/M phase, and reduced to 23.89% of cells in the S phase [47].

#### 2.1.3. EGR—Early Growth Response

The Early Growth Response gene (EGR-1) is activated by stress, injury, mitogens and differentiation [52]. This gene regulates the expression of other genes, which are involved in the control of growth and apoptosis such as: p21, p53, PTEN, Gadd45 [53].

Curcumin suppressed proliferation in human high-metastatic NSCLC cells 95D by EGR-1 in a dose-dependent manner. NSCLC cells transfected with EGR-1 siRNA notably inhibited EGR-1 expression, specifically siRNA3 [52]. Also, it has been found that curcumin inhibits human colon cancer cell growth via suppressing EGR-1 [54].

#### 2.1.4. β-Catenin

The β-catenin is located in three cellular pools (cell membrane, cytoplasm and nucleus), mainly in the cell membrane [55]. The main event of the activation of Wnt/β-catenin pathway is the nuclear translocation of beta-catenin, which binds to T-cell factor (TCF) in the nucleus [56]. The intracellular levels of beta-catenin are regulated by the phosphorylation of GSK-3β. Curcumin showed suppressing this phosphorylation in LNCaP prostate cancer cells, inducing the degradation of beta-catenin affecting the cell proliferation [56].

Curcumin suppressed cell growth by inhibiting the activation of Wnt/β-catenin pathway in desmoplastic cerebellar medulloblastoma (DAOY) cells. In this study, the expression of nuclear beta-catenin was significantly decreased; however, there was no effect on the expression of cytoplasmic beta-catenin levels. In addition, curcumin promote the activation of GSK-3β and its downstream target cyclin D1. The authors concluded that curcumin could be useful in the medulloblastoma treatment [57].

### 2.2. Protein Kinases

Protein kinases are a group of tyrosine or serine/threonine kinase enzymes whose function is to modify others proteins by attaching phosphate groups through the phosphorylation process. Tyrosine phosphorylation has a vital role in several important cellular pathways of eukaryote physiology, as well as in human diseases [58,59].

Protein kinases mediate most of the intracellular signal-transduction pathways in eukaryotic cells, control metabolism, transcription, mRNA processing, cell division, apoptosis and differentiation. Moreover, tyrosine phosphorylation mediated by protein kinases also regulate communication between neighboring cells, motility of cells and transport of molecules to within the cell [60,61]. Deregulation in tyrosine phosphorylation has been associated to a variety of cellular disorders and human diseases, such as cancer, diabetes, cardiovascular disorders, inflammatory diseases and immune deficiencies [62,63,64,65]. Specifically related to cancer, several studies have shown that deregulation of several protein kinases, including MAPK, Raf kinase, Akt, mTOR, MLK3, Src kinase, AMPK and protein kinase D are associated to a variety of cancers, such breast, gastric, thyroid, prostate, lung, liver and colorectal cancer [66,67,68,69,70,71,72,73,74].

#### 2.2.1. EGFR—Epidermal Growth Factor Receptor

Also known as ErbB1 or HER1, EGFR is a member of the ErbB family of receptors. The structure of EGF receptor is represented by an extracellular ligand-binding domain, a single transmembrane region with hydrophobic characteristics, and an intracellular module including the tyrosine kinase domain [75].

The EGFR pathway contributes in many ways to cancer proliferation and angiogenesis to many types of cancer. Curcumin decreased expression of EGFR, and also EGFR mRNA levels in bladder cancer cells [76].

An autophosphorylation activity of the EGFR tyrosine kinase have been observed after a short-term treatment of curcumin in dose and time dependent manner in human epithelial cancer cells (A431). Curcumin was able to inhibit EGFR tyrosine kinase in a concentration of 1 μM after 4 h of cell exposoure. The exact molecular mechanism of this short-term inhibition remains unknown [75].

#### 2.2.2. Polo-Like Kinase (PLK)

Polo-like kinases are important proteins on regulation of the cell cycle. It is related to spindle assembly, which has been found in high levels in colorectal cancer than normal colon tissues [77]. Curcumin downregulates PLK resulting in inhibition of the cell growth. It was characterized that curcumin promote cell cycle arrest in the G2/M phase and decrease the expression of some genes including tubulin genes and p53 related to colon cancer [78]. In some cancer cell lines, inhibition of PLK leads to cellular senescence correlating to the number of cells arrested in mitosis [79].

#### 2.2.3. Phosphatidylinositol 3-Kinase (PI3K) Pathway

PI3K is a protein that acts in the mechanism of cell survival. Its expression or activation is upregulated in diseases, such as diabetes and cancer. Akt is a mediator of PI3K signaling and affects directly the apoptosis process, targeting related proteins [80].

The influence of PI3K/Akt pathway and the effect of RES on cell growth were evaluated in different cancers cells. PI3K and MAPK are associated with HIF-1α accumulation and increase of VEGF expression, leading to angiogenesis [81]. In a study conducted to evaluate the influence of the RES in the accumulation of HIF-1α and VEGF expression in human tongue squamous cell carcinoma and hepatoma cells induced by hypoxia condition, it was observed that resveratrol was able to reduce the accumulation of HIF-1α and the expression of VEGF through inhibition of Akt and p42 and p44 MAPK phosphorylation [82].

In another study using human diffuse large B-cell lymphoma, it was observed that the resveratrol inhibited Akt phosphorylation following downstream targets, such as p70 S6K, S6 ribosomal and FOXO-3a. More specifically, it provides an improved comprehension of one possible mechanism of action, which involves the inhibition of PI3K pathway. This inhibitory effect exhibited a direct relationship with a decreased activity in the glycolysis pathway and may be the cause of cell cycle arrest in G0/G1 phase according authors observations [83].

The exposure of prostate cancer cells to resveratrol demonstrated that inhibition of the PI3K pathway reduces the phosphorylation of GSK-3 protein, which is related with the modulation of expression of cyclin D1, and decreases the activation NF-κβ [84,85].

#### 2.2.4. MAPK (p38 e ERK)

Resveratrol effects on MAPK are described in the literature. Using breast cancer cells, it was demonstrated that this polyphenol causes cycle cell arrest in S/G2M phase and upregulates the levels of phosphorylated p38 e ERK and increase p21 and p53R2 levels [86]. Another study using the same type of cancer cells also demonstrated the activity of resveratrol in the activation of p38. Resveratrol caused cycle cell arrest in G0/G1 phase. It also increased the activation of p38, p21 and p53 levels and decreased pRb hyperphosphorylated. Additionally, it was observed inhibition of ER expression, related to p53 activity. ER is described to play an important role in breast cancer cell proliferation [87].

### 2.3. Phosphodiesterases (PDEs)

Phosphodiesterases consist of a family containing 11 isoenzymes, which are responsible for hydrolyze two important second messengers that regulate cellular responses to external stimuli: the cyclic adenosine-3′,5′-monophosphate (cAMP) and the cyclic guanosine-3′,5′-monophosphate (cGMP).

These isoenzymes play an important role in cancer, and were found to be upregulated in angiogenesis and various types of tumors. For curcumin, it was found modifications in the pattern of PDE1A expression at transcriptional level. After curcumin treatment, the expression of PDE1A was dramatically reduced in B16F10 melanoma cancer cells. These findings indicate that PDE1A has an important role in the anti-proliferative effects of curcumin, and its inhibition may recover normal intracellular signaling contributing to the treatment [88]. Other isoforms (PDE2 and PDE4) were described to be upregulated in human umbilical vein endothelial cells (HUVECs). In these cells, the inhibition of PDE2 and PDE4 activities decrease the angiogenesis and cell proliferation [89].

### 2.4. Angiogenesis

Angiogenesis is involved in several biological processes. Nonetheless, its involvement in pathological processes, notably in tumor growth and metastasis still have been extensively investigated [90]. Some important pro-angiogenic and anti-angiogenic factors include: VEGF, MMPs, FGF (fibroblast growth factor) and HGF (hepatocyte growth factor). However, among these factors, VEGF and its receptors were described to be key regulators of both physiological and pathological vasculogenesis and angiogenesis [91,92].

VEGF is an important and multifunctional signaling glycoprotein that comprises a family of structurally related mitogens: VEGF-A, VEGF-B, VEGF-C, VEGF-D and placental growth factor (PIGF). These growth factors regulate a family VEGF receptors tyrosine kinases (VEGFR-1, VEGFR-2 and VEGFR-3) and promote endothelium regeneration, blood vessel regeneration and increase vascular permeability. However, VEGF-A (commonly known as VEGF) is the central member of the VEGF family and the majority of angiogenic effects related to these growth factor family are attributed to the interaction of VEGF-A with VEGFR-2 [93,94].

#### HIF-1/VEGF/bFGF

Cancer tumors activate hypoxia-inducible factor (HIF) under hypoxic conditions as a survival mechanism that ultimately leads to angiogenesis progression. It has been reported the effect of curcumin on vascular endothelial cells under hypoxic conditions using human umbilical vein endothelial cells (HUVECs). Specifically, curcumin downregulates HIF-1α protein and VEGF expression by blocking hypoxia-stimulated angiogenesis [95] and demonstrates anti-proliferative and anti-angiogenic properties [96].

During the tumor development, VEGF is a critical pro-angiogenic stimulator for neovascularization. The VEGF-VEGFR-2 complex is required to maintain a subset of vasculatures in healthy tissues and organs. Curcumin can block the VEGF-VEGFR-2 signaling pathways in HUVECs by suppressing the phosphorylation of VEGFR-2 induced by VEGF [97].

The effects of resveratrol against VEGF alter cell proliferation in endometrial cancer [98], myeloma [99], osteosarcoma [100], renal cancer [101] and melanoma [102]. High levels of VEGF were observed in endometrial carcinoma cells cultured in vitro under hypoxia conditions. However, after resveratrol treatment it was observed a reduced level of VEGF in a dose dependent manner, suggesting an anti-angiogenic activity when angiogenesis is induced under hypoxia [98].

The cellular viability of osteosarcoma cells and human renal cancer cells was evaluated in the presence of resveratrol. It was observed a dose dependent inhibition of growth in both cells, with no detectable VEGF and VEGF mRNA even at high doses of resveratrol (up to 40 μmol/L) [100,101].

Resveratrol also inhibited in a dose dependent manner the proliferation, migration and tube formation of HUVEC induced by co-culture with myeloma cell. In order to comprehend the mechanism that resveratrol acts in angiogenesis, it was determinate the levels of VEGF, basic fibroblast growth factor (bFGF) and metalloproteinases 2 and 9 (MMP-2 and MMP-9) [99]. Interestingly, it was found that resveratrol inhibited the expression of VEGF and bFGF, besides to suppress the expression of MMPs, which may explain its effect in the angiogenesis [99].

Additionally, studies to characterize the antiangiogenic effect of RES were evaluated in a chick chorioallantoic membrane (CAM) model. Resveratrol reduced the angiogenesis in the membrane induced by fibroblast growth factor-2 (FGF-2). Moreover, the tumor growth in the CAM model was inhibited, as well as, the angiogenesis. The level of p53 was quantified and a significant reduction was determinated after treatment using resveratrol. This results suggest an apoptotic effect induced by resveratrol, which might be responsible to stop tumor growth and angiogenesis [103].

### 2.5. Cell Cycle Regulators

The cell cycle is divided into four main phases: G1-S-G2-M. The G1 phase, also known as GAP 1, is the first growth stage of the cell cycle. During the S (synthesis) stage, the chromosomes of somatic cells are replicating. The G2 phase (GAP 2) is the final sub-phase of interphase in the cell cycle, prior to mitosis (M phase) [104].

Cyclin B1 is overexpressed in many tumors and is needed to forward cells from G2 phase to M phase during the cellular cycle. It was demonstrated that after 24 h of curcumin treatment, protein and mRNA levels of cyclin B1 were downregulated. In addition, flow cytometry data have shown arrested effect on cell cycle involving G2/M phase in small cell lung cancer (SCLC) cells [105].

Curcumin inhibits cyclin-dependent kinase 2 (CDK2) activity in vitro and decrease the proliferation of colon cancer cells, indicating G1 cell cycle arrest in a dose-dependent manner. The percentage of sh-CKD2-transfected HCT116 colon cancer cells in G1 phase was higher after curcumin treatment that those of control groups. Computational molecular docking studies have demonstrated a very good binding affinity between CDK2 and curcumin with a score of −12.69 kcal/mol, validating previous in vitro data [106].

Resveratrol has been described to cause cell cycle arrest in different types of cancers, mainly at low concentrations. Cycle cell arrest between the G1 and S phases were observed in prostate cancer cells [107], pituitary prolactinoma [108], human epidermoid carcinoma [109] and lung cancer cells [110]. Similar results were found in these studies, showing that resveratrol decreased the levels of cyclins (D1 and D3) and of CDK (4 and 6). In addition, resveratrol increased the expression of p21 and p27.

Furthermore, the inhibition of cell proliferation of pituitary prolactinoma cells, an estrogen-dependent tumor, caused by resveratrol persists after the end of the exposure of this compound, which indicates an irreversible suppressive effect [108]. The phosphorylation of pRb was inhibited in two different type of cells exposured to resveratrol [108,109]. Resveratrol was described to inhibit kinases, therefore, authors assumed that a reduction of cyclin D1 levels could be associated with this effect [109].

The exposition of hepatocarcinoma cells to resveratrol induces cell accumulation in S phase, by a reversible process. Regarding cell cycle regulators, it was observed reduction in the levels of cyclin D1 and p21. However, the levels of phosphorylated CDK2 and Chk2 have been increased. PI3K pathway may be related, in part, with cell cycle arrest in S phase [111].

In addition, it was observed that resveratrol treatment of oral squamous carcinoma cells resulted in cell cycle arrest in G2/M phase. It was also observed an increase in cyclin A and B levels, possibly related to the high expression of protein kinase Myt-1 [112].

### 2.6. SIRT

Sirtuin family is composed by seven sirtuins types, defined as NAD^+^-dependent histone deacetylases. SIRT-1 is responsible for deacetylation of transcriptional factors, DNA repair proteins and signaling factors. It regulates important biological activity, including cell survival, gene expression, metabolism and senescence [113].

Resveratrol has been described as a potential SIRT activator, since this compound inhibited cell proliferation in a SIRT-1 dependent way. In this study, the anti-proliferative effect of this compound was studied only in gastric cancer cells that could express SIRT-1. It was observed that resveratrol treatment caused a G1 phase arrest, decrease the levels of cyclin D1, CDK4 and CDK6 and increase the levels of p21. In knockout cells that can express SIRT-1, resveratrol was not capable to inhibit cell proliferation [114].

Similary, in a study using breast cancer cells, resveratrol inhibited cell proliferation by stimulating SIRT-1. Activation of AMPK pathway leads to mTOR activation, which stimulates the cell proliferation. It was observed that resveratrol can block AMPK phosphorylation by SIRT-1 activity overexpressed in tumor cells [115].

The effects of resveratrol on cell proliferation of hepatocarcinoma cell under high concentration of glucose were evaluated in another study. The results showed that high glucose concentration upregulated activated STAT-3 and enhanced cellular viability. Resveratrol was able to suppress proliferation and activation of STAT-3 and Akt [116].

### 2.7. Others Targets

Others proteins, enzymes, and transcription factors involved in cell proliferation and described as target for curcumin and resveratrol are described in Table 1 and Table 2.

## 3. Metastasis

Although several advances have been achieved in the last years against cancer, the mortality rate related to metastasis is still about 90% [133,134,135]. Therefore, cellular pathways involved in metastasis have been extensively described as promising therapeutic target for a variety of cancers [136,137,138]. Metastasis is the spread and growth process of solid cancers cells from the original neoplasm to distant organs through several cellular mechanisms, such as angiogenesis, invasion and proliferation [139,140]. The process involved in metastasis is fairly complex and begins when primary cancer cells break away from their original tumor environmental and invade through the basement membrane reaching the circulation. Subsequently, these metastasizing cells will reach and settle microenvironment in distant organs [141]. This metastatic progression depends on several biochemical, genetic and epigenetic factors in the original tumor cells and association to the new microenvironment [142].

**Figure 2:**
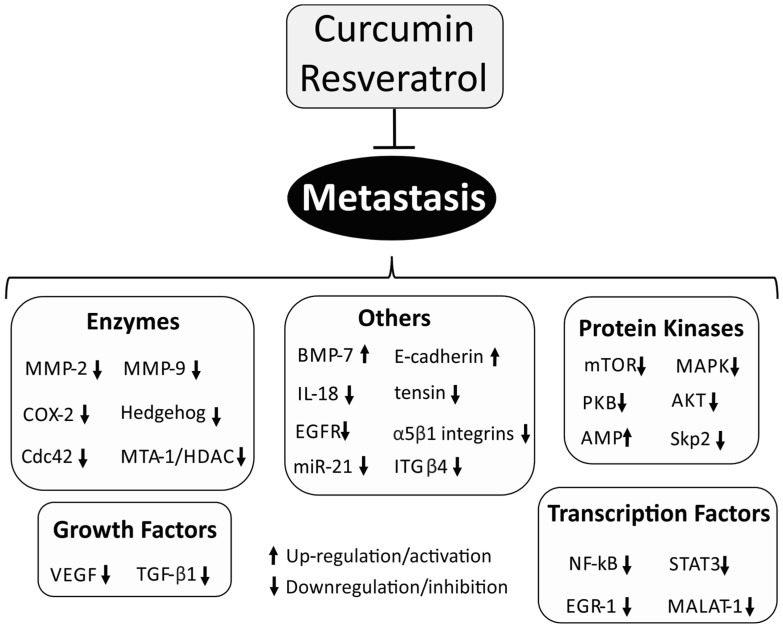
The control of metastasis by curcumin and resveratrol.

Curcumin and resveratrol modulate many of these cellular pathways, including transcription factors, proteins, enzymes and growth factors (Figure 2) [143]. Although the precise mechanism of action of polyphenols remains unclear, several studies have highlighted the inhibitory effect of these compounds in a number of molecular targets and signaling pathways involved in cancer metastasis [144,145,146,147]. In this section, we highlighted the major cellular targets involved in metastasis that curcumin and resveratrol have the ability to modulate.

### 3.1. NF-κB Signaling Pathway

Curcumin is able to modulate NF-κB signaling pathway directly and indirectly by downregulation or upregulation some key factors. Aggarwal and coworkers demonstrated that curcumin inhibited tumor cell invasion through inhibition of I-κB kinase complex (IKK) and protein kinase B (Akt) in human myeloid leukemia and human embryonic kidney cells. The inhibition of IKK and Akt blocks the phosphorylation of p65, which led to a suppression of cellular events required for NF-κB gene expression. As a result, the inhibition of NF-κB by curcumin resulted in downregulating of several NF-κB-regulated gene products involved in cellular proliferation and metastasis including COX-2, cyclin D1, c-myc, MMP-9, VEGF and intercellular adhesion molecule-1 [148].

Similarly, it was also demonstrated that curcumin inhibits translocation of NF-κB from the cell nucleus by inhibition of the I-κB kinase complex in both, breast and prostate cancer cells [149,150]. The authors have demonstrated that inhibition of NF-κB activity reduces the expression of inflammatory cytokines, such as, CXCL1 and CXCL2. Some cancer cells with potential to metastasize to lung overexpress these inflammatory cytokines and promotes infiltration of inflammatory cells, which lead to angiogenesis and metastasis process [151]. Moreover, in vivo experiments using mice demonstrated that curcumin was able to reduce the number of lung metastases formed from circulating prostate cancer cells after 35 days of treatment [150].

In fact, several studies have demonstrated the narrow relationship between curcumin and NF-κB signaling pathway in cancer metastasis. Narasimhan and Ammanamanchi have shown that curcumin was able to block the invasion of breast carcinoma cells using a matrigel invasion experiment. They have concluded that curcumin reduced the expression and transcriptional activity of NF-κB p65 protein and decreased the levels of the Recepteur d'Origine Nantais tyrosine kinase (RON) [152]. RON plays an important role in cell proliferation, differentiation and metastasis. Its overexpression in patients with breast cancer is associated to a poor prognostic [153].

Zong and colleagues also demonstrated the potential therapeutic application of curcumin to inhibit metastatic progression of breast cancer cells. They investigated the urokinase-type plasminogen activator (uPA), a serine protease protein that plays an important role in tumor growth and metastasis. The authors found that curcumin was able to reduce uPA expression through downregulating NF-κB activity [154].

In a different work, the inhibition of the human astroglioma cells invasion and metastasis was reported for curcumin. The authors proposed that mechanism of action involves the downregulation of NF-κB, which resulted in an inhibition of matrix metalloproteinase-9 [155]. Interestingly, an in vivo study using human prostate adenocarcinoma LNCaP xenograft cells demonstrated that curcumin was able to reduce metastatic process in mice though inhibition of NF-κB activity leading to a reduction in the expression of its related genes, including VEGF, Bcl-2, Bcl-XL, uPA, cyclin D1, MMP-2, MMP-9, COX-2 and IL-8 [156].

By the other hand, the activity of resveratrol against NF-κB during metastasis is also described by several groups. Chen and colleagues have reported that resveratrol successfully inhibited epithelial-mesenchymal transition in mouse melanoma model and reduced cancer migration and metastasis. The authors concluded that resveratrol downregulated NF-κB activity and influenced in epithelial-mesenchymal transition [157]. In another study, it was demonstrated that resveratrol was able to block the migration and invasion of human metastatic lung and cervical cancer cells. Resveratrol inhibited the activity of NF-κB and AP-1 leading to reduction in MMP-9 expression [158]. Liu and coworkers also demonstrated the effect of resveratrol on NF-κB inhibition and its downstream events in human lung adenocarcinoma cell metastasis [159].

Heme oxygenase 1 (HO-1) is an important enzyme involved in angiogenesis and tumor metastasis and its activity have been associated to matrix metalloproteinases expression [160]. Resveratrol suppressed NF-κB activity leading to inhibition of HO-1 and subsequently downregulating the expression of MMP-2 and MMP-9 in lung cancer cells [159]. Resveratrol was also reported acting as an inhibitor of cancer invasion and metastasis of human hepatocellular carcinoma cells. The authors have demonstrated that resveratrol suppressed TNF-α-mediated MMP-9 expression through downregulation of NF-κB signaling pathway activity [161].

Ryu and coworkers have reported the antimetastatic activity of resveratrol in human glioma cancer cells induced by TNF-α overexpression. Resveratrol suppressed NF-κB activation and downregulated the expression of urokinase plasminogen activator (uPA), thereby leading to a reduction of TNF-α-induced cell invasion [162]. Adhesion molecules, such as intracellular adhesion molecule-1 (ICAM-1), vascular cell adhesion molecule-1 (VCAM-1), E-cadherin and E-selectin plays a central role in endothelial adhesion of a number of cancer cells and are closely related to cancer invasion and metastasis [163,164]. Therefore, the inhibition of cellular pathways related to adhesion molecules have been considering as a promising anti-metastasis target [165]. Park and colleagues have demonstrated the anti-metastatic activity of resveratrol in human fibrosarcoma cells. Resveratrol blocked cancer cell adhesion to endothelial cells through inhibition of ICAM-1 expression; however, they observed that this downregulation of ICAM-1 expression was due to suppression of NF-κB activation. Therefore, indirectly the inhibition of NF-κB pathway has an important role in ICAM-1 expression [166].

### 3.2. Matrix Metalloproteinase (MMP)

Matrix metalloproteinases (MMPs), collectively called matrixins, represents a group of enzymes with proteolytic activity that exist in the extracellular matrix (ECM) and are involved in most of the physiological conditions, including embryogenesis, reproduction, organ development, wound healing, angiogenesis and apoptosis [167,168]. These zinc-dependent endopeptidases also plays a vital role in the spread and dissemination of cancer and are closely related to tumor metastasis process [169]. The proteolytic activity of MMPs involves the ECM degradation and evidences have shown that the expression of specific MMPs, such as MMP-2 (Gelatinase A) and MMP-9 (Gelatinase B), are associated with a wide range of human cancers [170,171,172,173].

Several studies have shown the potential use of curcumin in cancer metastasis by reducing the expression and activity of matrix metalloproteinases. Chen and colleagues have demonstrated that curcumin suppressed migration and invasion of human endometrial carcinoma cells. Curcumin successfully reduced the expression of MMP-2 and MMP-9 through downregulation of the extracellular signal regulated kinase (ERK) signaling pathway [174]. This protein kinase is involved in the biosynthesis of MMP and plays a vital role to regulate the proliferation and invasion of endometrial carcinoma cells [175]. Another study demonstrated that curcumin also suppress the tumor growth and metastasis in prostate cancer cells by inhibition of MMP-9. Furthermore, curcumin also inhibited the expression of cellular matriptase, a membrane-anchored serine protease that is associated to a number of tumors with poor prognosis [176].

Indeed, MMP-2 and MMP-9 are the main enzymes associated with metastasis whose activities are inhibited by curcumin. This inhibitory activity may occur through different pathways. For instance, it was demonstrated that curcumin inhibited lung cancer cells invasion by modulating the PKCα/Nox-2/ROS/ATF-2 signaling pathway leading to downregulation of MMP-9 expression. During the metastasis process, the activation of MMP-9 gene promoter enhances MMP-9 transcription [177]. Another study pointed out that Rac1/PAK1 pathway is a promising target in MMPs activation pathway. The authors have demonstrated that curcumin reduces lung cancer cell metastasis through inhibition of MMP-2 and MMP-9 expression mainly by downregulation of Rac1/PAK1 [178]. Banerji and coworkers demonstrated the effect of curcumin on MMP-2 activity in murine melanoma cells. They observed a reduction in membrane type-1 matrix metalloproteinase (MT1-MMP) and focal adhesion kinase (FAK) production, leading to a reduction of MMP-2 expression after 15 days of curcumin treatment [179]. FAK and MT1-MMP plays a vital role in intracellular signaling pathway and studies have associated its activity to MMP expression [180,181]. Further, the same research group has demonstrated that curcumin was able to reduce tumor cell invasion and metastasis in human laryngeal squamous carcinoma cells. The authors suggested that curcumin inhibited MMP-2 expression through modulation of FAK and MT1-MMP signaling pathway [182]. Liao and colleagues also demonstrated the inhibitory effect of curcumin in MMP-2 expression on lung cancer cells due to downregulation of the expression of glucose transporter 1 (GLUT-1) and MT1-MMP [183].

For resveratrol, studies have demonstrated its anti-metastatic effect against several types of cancers by downregulation of MMP expression and its enzymatic activities, mainly MMP-2 and MMP-9. Among the types of cancer that resveratrol was active, we included glioblastoma [184], breast [185,186], multiple myeloma [99,187] and hepatocellular carcinoma [188].

### 3.3. E-Cadherin

The epithelial cell–cell adhesion molecule cadherin 1, also known as epithelial cadherin (E-cadherin) is a transmembrane glycoprotein that mediates cell-cell adhesion through calcium-dependent binding between two E-cadherin molecules at surface of adjacent cells [189,190]. E-cadherin is essential for the epithelial cell behavior and evidence have shown that loss of its function is associated with the proliferation of a number of cancers, including lung [191], pancreatic [192], oral [193], liver [194], gastric [195], prostate [196] and ovarian [197]. The cellular function of E-cadherin depends on the interaction with the catenin protein family, such as α-, β- and p120 catenins [198]. β-catenin is a key cytoplasmic protein that acts in association with α-catenin and creates a link between E-cadherin and the actin cytoskeleton [189,199].

Chen and colleagues described the cell invasion and metastasis inhibitory activity of curcumin in a mice lung cancer [200]. Specifically, curcumin up-regulated the expression of E-cadherin through activation of the tumor suppressor DnaJ-like heat shock protein 40 (HLJ1), which has been associated with cell proliferation, invasion and metastasis against a variety of human cancers [201]. The authors also suggested that curcumin modulates HLJ1 by enhancing the JNK/JunD expression [200]. Further, the same research group demonstrated the anti-metastatic effect of curcumin against colorectal cancer cells using in vivo assays [202]. Curcumin played its activity by upregulation of E-cadherin expression leading to an inhibition of mesenchymal transition (EMT). EMT-related genes has been associated with cancer progression and metastasis [203]. Likewise, not only E-cadherin overexpression was observed for curcumin activity, but also the suppression of Sp-1 transcriptional activity and the inhibition of focal adhesion kinase (FAK) phosphorylation [202]. Curcumin was able to block papillary thyroid cancer cells migration and invasion in a dual pathway, by increasing E-cadherin expression and inhibition of MMP-9 activity [204,205,206]. Zhang and coworkers have shown the potential application of curcumin in reducing progression and metastasis of colon cancer cells through the overexpression of E-cadherin. Moreover, the authors demonstrated that others signaling pathways were involved, including downregulation of vimentin, inhibition of Wnt signaling pathway and downregulation of CXCR4 [207].

### 3.4. Protein Kinases

Du and colleagues have reported the effect of curcumin in the inhibition of cancer invasion and metastasis in human prostate-associated fibroblasts. Curcumin suppressed the MAOA/mTOR/HIF-1α signaling pathway thereby leading to a downregulation of reactive oxygen species (ROS), CXC chemokine receptor 4 (CXCR4) and interleukin-6 (IL-6) receptor, which has been associated to migration of prostate carcinoma cells [208]. The inhibition of the Akt/mTOR/P70S6K kinase-signaling pathway by curcumin was also reported in human melanoma cells. Curcumin reduced the phosphorylation of this kinase-signaling pathway leading to an inhibition of cell invasion. The authors have demonstrated that curcumin was able to reduce melanoma growth against an in vivo melanoma model [209].

Guan and coworkers have reported the antiproliferative and antimetastatic activity of curcumin in breast cancer cells. They concluded that for these cells, curcumin increased AMP-kinase phosphorylation leading to a reduction of Akt protein expression and subsequently cell migration suppression [210].

Another study has demonstrated that curcumin inhibited cell growth and invasion through downregulation of S-phase kinase associated protein 2 (Skp2)-pathway in glioma cancer cells. The authors concluded that the suppression of Skp2 activity promotes an upregulation of p57 [123], which acts as an regulator of apoptosis, differentiation and migration in tumorigenesis and its inhibition is related to tumor growth [211].

Mitogen-activated protein kinase (MAPK) pathway comprises a family of protein kinases, including extracellular-signal regulated kinases (ERK), c-Jun *N*-terminal Kinase (JNK) and p38 MAPK. These protein kinases plays an important role in the regulation of genes involved in cell migration and invasion [212]. Several in vitro and in vivo studies have reported the anti-metastatic activity of resveratrol through downregulation of MAPK pathways against cancers, such as ovarian [213,214], oral [215], breast [216,217], fibrosarcoma [218], hepatocellular carcinoma [219] and osteosarcoma [220].

Akt/protein kinase B (PKB) is another important serine/threonine kinase that plays a central role in many signaling pathways involved in cell growth, proliferation and tumorigenesis, such as PI3K, PTEN, NF1, LKB1, TSC2, FOXO and eIF4E [221,222]. Resveratrol have been described as an inhibitor of the Akt signaling pathway in a number of human cancer, including cutaneous melanoma [223], glioblastoma [224], pancreatic [225], and breast [226]. In most cases, the inhibition of this pathway leads to a reduction in MMP expression, and consequently inhibition of cancer invasion and metastasis.

### 3.5. Vascular Endothelial Growth Factor (VEGF)

Kalinski and colleagues have reported the angiogenesis and anti-metastatic activity of curcumin in human chondrosarcoma cells. Curcumin inhibited interleukin-1 (IL-1) signaling by blocking the recruitment of IL-1 receptor associated kinase (IRAK) to the IL-1 receptor. IL-1 plays a central role in inflammatory, immune and malignant processes and its downstream events are associated with activation of NF-κB and metastasis-related genes, such as, VEGF-A [227]. Curcumin was also described with anti-metastatic activity through mice gastric cancer model. The authors reported that curcumin downregulated the expression of vascular endothelial growth factor receptor 3 (VEGFR-3) and its mRNA, prospero homeobox 1 (Prox-1) and podoplanin. This compound leads to a suppression of lymphatic vessel density, which is associated with poor prognosis in gastric cancer [228].

### 3.6. Hedgehog Signaling Pathway

The Hedgehog signaling pathway is an important family of proteins recognized for its importance in a number of cellular events including, proliferation, survival and differentiation [229]. Cumulative evidence strongly suggests its regulatory effect in the development of cancer angiogenesis and metastasis by modulating the expression of central proteins and transcription factors involved in cancer invasion, such as Snail protein, E-cadherin, angiogenic factors, cyclins, anti-apoptotic and apoptotic genes [230,231].

It was demonstrated the effect of resveratrol on metastatic prostate cancer cells by modulating the Hedgehog pathway. The authors have demonstrated that resveratrol-treated cells resulted in inhibition of epithelial-mesenchymal transition, exhibited an enhancement of E-cadherin expression and reduction of vimentin expression. In addition, resveratrol inhibited the expression of the transcription factor glioma-associated oncogene homolog 1 (Gli-1) [232], which plays an important role in the downstream events upon Hedgehog activation [233]. Gao and colleagues also demonstrated the anti-metastatic activity of resveratrol against gastric cancer cells by modulation of the Hedgehog signaling pathway through downregulation of Gli-1 expression. Moreover, resveratrol upregulated the expression of E-cadherin gene, decrease Snail protein and N-cadherin expression [234].

In different study, the role of Hedgehog pathway was once again described. Authors have found that the beneficial effect of resveratrol in the inhibition pancreatic cancer cells migration and invasion by suppression of this signaling pathway. Resveratrol was able to reduce Gli-1 expression and hypoxia-induced reactive oxygen species production leading to a downregulation of Hedgehog activity and thereby inhibiting the cell invasion. Furthermore, resveratrol also inhibited HIF-1α, uPA and MMP-2 expression [235].

### 3.7. STAT-3 Signaling Pathway

Signal transducer and activator of transcription-3 (STAT-3) is a transcription factor that belongs to the STAT protein family [236]. This signaling pathway is present in cytoplasm in their inactive state and upon activation-dependent tyrosine phosphorylation; this transcription factor translocates into the cell nucleus and binds to specific enhancer elements for transcription process initiation. A number of stimuli are known to activate STAT-3 pathway, including cytokines, growth factors and oncogenic proteins. Currently, there is cumulative evidence that point out its important role in metastasis process of a variety of human cancers, such as leukemias, lymphomas, head and neck, breast, lung, gastric, hepatocellular, colorectal and prostate cancers [237]. STAT-3 target genes are involved in several cellular events related to cancer metastasis, such as invasion, cell survival, angiogenesis and tumor-cell immune evasion [238].

Lee-Chang and coworkers have reported the in vivo anti-metastatic activity of resveratrol against metastatic lung cancer. The authors described that resveratrol downregulates STAT-3 activity and reduces the tumor-evoked regulatory B cells (tBregs) production and activity [239]. tBregs is thought to be an important mediator in the protection of metastatic cancer cells by modulation of CD4+ T cells to inactivate antitumor NK cells and the effector CD8+ T cells conversion [240].

Resveratrol was also reported as an inhibitor of tumor growth and metastasis against tumor-associated macrophages. The mechanism seems to be through inhibition of lymphangiogenesis and M2 macrophage activation and differentiation [241]. M2 macrophage activation has been associated to tumor growth and metastasis in tumor-associated macrophages [242]. The authors demonstrated the inhibitory effect of resveratrol on STAT-3 phosphorylation during M2 macrophage differentiation. This effect blocks the differentiation process, decreases VEGF-C-induced migration/invasion, and capillary-like tube formation in lymphatic endothelial cells by modulation of IL-10, MCP-1 and TGF-β1 [241]. Wang and colleagues also reported the inhibitory effect of resveratrol in the STAT-3 phosphorylation in human glioblastoma cells leading to a reduction of hypoxia-induced migration and invasion [243]. Mechanistically, resveratrol inhibited cancer metastasis through upregulation of microRNA-34a activity, which act as an important tumor suppressor and is downregulated by STAT-3 [243,244].

### 3.8. Others

For resveratrol and curcumin, not only those mechanisms described above are responsible to inhibit the metastasis process, but different biochemical signaling pathways has shown an important contribution to modulate this process as well. For instance, Chen and colleagues reported the effect of curcumin to prevent cancer progression and metastasis using an in vivo lung cancer model. In this work, it was demonstrated that curcumin downregulated the expression of Cdc42 and Rho GTPase protein that plays an important role in proliferation, invasion and metastasis [245]. In fact, several studies have associated the overexpression of Cdc42 and the progression of a variety of human cancers [246]. The same research group has demonstrated the anti-metastatic activity of curcumin in non-small cell lung cancer by decreasing the expression of early growth response protein 1 (EGR-1), and thereby reducing the adherens junctions and Wnt signaling pathway activity. This signaling pathway is essential for cancer cells detach from the epithelium and achieve metastasis to distant tissues [52].

Integrin β4 (ITG β4) is a heterodimeric transmembrane receptor that act as structural link between cells or cells to the extracellular matrix. Cumulative evidences reveal that ITG β4 is associated in several signaling pathways leading to a variety of cellular events, including cell apoptosis, differentiation, cancer invasion and metastasis [247]. It was demonstrated that curcumin successfully inhibited the palmitoylation process of ITG β4 in breast cancer cells. This process is a post-translational modification and it is essential for ITG β4 signaling activity that promote a reduction in cancer invasion [248].

Dorai and coworkers have reported the anti-metastatic activity of curcumin in bone cancer. Curcumin was able to inhibit metastasis process from bone cancer to prostate using an in vivo model. The authors suggested that curcumin upregulated the bone morphogenic protein-7 (BMP-7), which act as a metastasis inhibitory protein and its upregulation promoted a modulation of transforming growth factor-β (TGF-β) function [249]. TGF-β plays a vital role in the cycle of bone metastasis. Studies have shown that its binding with BMP-7 leads to increased expression of E-cadherin and therefore, the inhibition of bone cancer metastasis [250].

Curcumin also inhibited in vivo tumor progression and metastasis in colorectal cancer. The study concluded that curcumin reduced miR-21 transcriptional regulation and expression through inhibition of activator protein-1 (AP-1) [251]. miR-21 is a microRNA that plays an important role in cellular proliferation, differentiation and apoptosis and studies have associated its overexpression in a variety of human cancer, including glioblastoma, ovarian carcinoma, hepatocellular carcinomas, head and neck cancer and chronic lymphocytic leukaemia [252]. In another study, curcumin suppressed migration of cancer glioma cells by decreasing miR-21 expression [253].

Phosphatase of regenerating liver-3 (PRL-3) is a tyrosine phosphatase and cumulative evidence have associated its overexpression with a number of human cancer metastasis [254,255]. Wang and collaborators have demonstrated that curcumin inhibits in vivo metastasis through downregulation of PRL-3 expression in melanoma cells. Specifically, the inhibition of PRL-3 cause a reduction of Src and STAT-3 phosphorylation [256].

Several others proteins, enzymes, and transcription factors have been described as a target for resveratrol leading to inhibition of cancer metastasis. Some examples reported in the literature are presented in Table 3.

## 4. Cellular Death

### 4.1. Apoptosis

An important event in the intrinsic apoptotic pathway, or mitochondrial pathway, is the change in mitochondrial membrane potential that leads to an increase in permeabilization of the outer mitochondrial membrane and the release of the proteins found in the space between the inner and outer mitochondrial membranes. The regulation of this permeabilization is coordinated by proteins of the Bcl-2 family and others components [266]. Bcl-2 is an antiapoptotic protein inserted in the outer of mitochondrial membrane. It has your antiapoptotic properties by regulating the activity of Bax and Bak, for example. These two proteins are able to move to the mitochondria, disrupt the function of Bcl-2, allow the permeabilization of the outer mitochondrial membrane and release the content of the intermembrane space [267].

Cytochrome c is an example of the released content of the mitochondrial intermembrane space. Once in the cytosol, cytochrome c binds to the *C*-terminal region of Apaf-1 (apoptotic protease activating factor-1), a cytosolic protein with an *N*-terminal caspase-recruitment domain (CARD), a nucleotide-binding domain and a *C*-terminal domain [268]. The association of dATP with Apaf-1 is facilitated by this binding and exposes its *N*-terminal CARD, which now is able to oligomerize and become a platform on which the initiator caspase-9 is activated through a CARD-CARD interaction [269]. This complex is called apoptosome and it is the responsible for caspase-3, that it is able to induce apoptosis [270,271].

**Figure 3:**
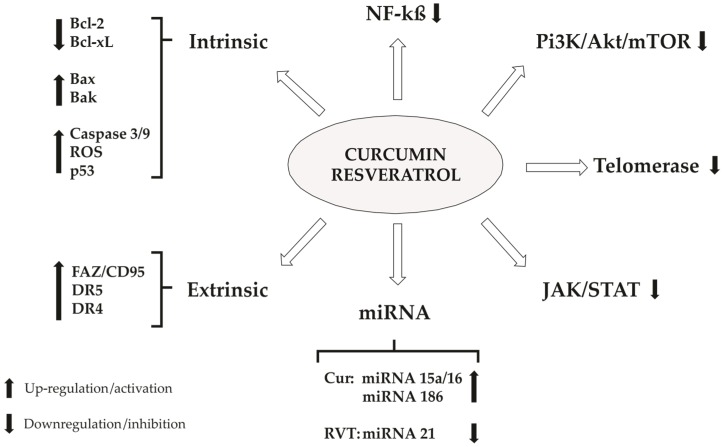
General scheme about curcumin and resveratrol effects in apoptosis.

Smac/DIABLO and Omi/HtrA2 are two others examples of the released mitochondrial proteins. They facilitate caspase activation by inhibiting the IAPs (inhibitor of apoptosis proteins), an endogenous inhibitor of caspases [272]. XIAP, cIAP1, cIAP2, survivin and livin (ML-IAP) are examples of IAPs. AIF (apoptosis inducing factor) is another protein of the mitochondrial intermembrane space that induces apoptosis caspase-independent. After an apoptotic insult, AIF translocate to the nucleus and induces chromatin condensation and DNA fragmentation. On the other hand, an overexpression of Bcl-2 blocks the AIF redistribution, inhibiting this apoptotic pathway [273]. A general scheme about apoptosis is presented in Figure 3.

The ability of resveratrol to direct target mitochondria was shown in bladder cancer cells and neuroblastoma cell lines. Experiments with intact cancer cell and isolated mitochondria were run and both of them resulted in a loss of mitochondrial membrane potential. Thus, it was shown that resveratrol was able to induce the release of cytochrome c and Smac/diablo in the intact cancer cell. An interesting result came from the neuroblastoma cell lines, which demonstrated that isolated mitochondria cytochrome c was not able to be released, indicating that the cytoplasmic content is important for this process [274,275].

In breast cancer cells [276] and glioma cells [277], resveratrol has demonstrated potential to activate caspase-3 and increase its activity. In breast cancer cell study, the cleavage of caspase-3 into its active form was observed. In addition, the role of caspase-3 in apoptosis was tested using a caspase-3 inhibitor, resulting in a decrease of cell death. Beyond that, in glioma cells study was also demonstrated the induction in the caspase-3 mRNA expression.

In human lung adenocarcinoma, has been demonstrated that the resveratrol-induced apoptosis is predominantly via intrinsic pathway and caspase-independent. It was demonstrated that in these cells AIF is the protein released from mitochondria. Also, resveratrol was able to induce Bak, but not Bax, activation and, when the first one is silenced, the release of AIF is prevented and the apoptosis is inhibited, indicating that Bak has an essential role in this caspase-independent AIF signaling pathway [278,279].

#### 4.1.1. ROS

Curcumin is capable to activate antioxidant enzymes, such as, glutathione-*S*-transferase (GST), quinine reductase and hemeoxygenase-1 [280]. There are a lot of works demonstrating that apoptotic induced effect by curcumin is due to reactive oxygen species (ROS) formation. It was reported that both papillary thyroid cancer cell line and cutaneous T cell lymphoma cells have a previous increased levels of ROS that is responsible to promote loss of mitochondrial membrane potential (MMP). These deregulations culminated in Bcl-2 reduction, cleavage of poly ADP-ribose polymerase (PARP) and apoptosis induction [281,282].

Curcumin has increased the levels of ROS and superoxide radicals (SOR) against human lung adenocarcinoma epithelial cells, leading to high levels of lipid peroxidation. They described that the antioxidant agent—*N*-acetyl cysteine—has prevented curcumin-induced ROS formation and apoptosis. They suggested that ROS formation induced by curcumin was able to activate the apoptosis in these cells [283].

In diffuse large B cell lymphoma cells lines (DLBCL) was demonstrated that resveratrol-induced apoptosis is related to release of ROS (reactive oxygen species). In a sequence of events, the ROS released is able to inactive Akt and FOXO1, GSK3 and Bad. Inactivated Bad allows a change in Bax protein conformation, which leads to variations in mitochondrial membrane potential, release of cytochrome c and apoptosis via intrinsic pathway. Moreover, ROS release also results in up-regulation of DR5, a death receptor, which increased the apoptosis in DLBCL, demonstrating, in this cell, that resveratrol is able to induce apoptosis via intrinsic and extrinsic pathway [284].

In SGC7901 cells, resveratrol was able to induce apoptosis and developed a pro-oxidant role, inducing the generation of reactive oxygen species. A treatment of this cells with a scavenger eliminated the pro-apoptotic effect of resveratrol, indicating that the pro-oxidant role of this polyphenol is essential for the apoptosis [285].

#### 4.1.2. Calcium Homeostasis

Calcium also appears to be an important role in apoptosis induces for curcumin. This polyphenol promoted apoptosis in color cancer cells through the increase in [Ca^2+^] and ROS formation. These effects promote a reduction in MMP and generate caspase-3 activation. The use of an intracellular calcium chelator promote a reversion in apoptosis [286]. A similar result was observed in human leukemia cells and was also verified that the caspase-3 inhibitor (z-VAD-fmk) was capable to block curcumin-induced apoptosis [287].

In a different study, the levels of ROS and intracellular [Ca^2+^] increased by curcumin have shown an important contribution to cause apoptosis. The use of the mitochondrial uniporter inhibitor (RU-360) partially suppressed curcumin-induced apoptosis. Moreover, the use of SKF-96365, a store-operated Ca^2+^ channel blocker, blocked the elevation of mitochondrial calcium, promoting a potentiation in curcumin-induced apoptosis [288].

Using human hepatocellular carcinoma J5 cells, it was also demonstrated for curcumin the ability to induce apoptosis through Ca^2+^-regulated mitochondria-dependent pathway. In vitro assays have demonstrated an increased level of cytoplasmatic cytochrome c, corroborating with reduced mitochondrial membrane potential hypothesis. Once again, for these cells it was observed an increase in ROS formation and cytoplasmic calcium accumulation. BAPTA, an intracellular calcium chelator, was capable to reduce curcumin-induced apoptosis, suggesting that this process is calcium dependent in these cells lines [289].

In mesothelioma cells (REN cells), resveratrol was able to induce a transient intracellular [Ca^2+^] elevation possibly by T-type Ca^2+^ channels. Experiments were run toward to Cav 3.2 isoform of this channel because its shown to be highly expressed in REN cells. The results have demonstrated that it is the major responsible for Ca^2+^ entry. Besides, Cav 3.2 siRNA inhibited the effect of resveratrol, which indicates the role of this channel. A comparison between normal cells and mesothelioma cells was studied and a difference in the peak levels of calcium have demonstrated a higher sensibility of cancer cells to resveratrol-induced changes. Furthermore, in cancer cells resveratrol was able to inhibit proliferation whereas in normal cells it was ineffective [290].

#### 4.1.3. Bcl-2 Family

In follicular lymphoma cell lines, curcumin inhibited the cellular proliferation and induced apoptosis through the increase in bcl-2 family proteins. The authors demonstrated a reduction in Bcl-xL levels for all cell lines. In addition, they characterized cell line-dependent changes in the level of Mcl-1, bcl-w, Bak, and Bok. All these process promotes increased levels of ROS. Curcumin also increase the lysosomal membrane permeability [291].

Similar observations were made for other cancer cell lines, including glioblastoma, colorectal, lung and endometrial carcinoma [292,293]. In human prostate cancer cells, it was observed reduction of pro-apoptotic proteins and induction of caspase 3 and PARP cleavage [294]. Yu and Shah (2007) verified through transfected human endometrial adenocarcinoma HEC-1-A cells the possibility of proto-oncogene Ets-1 promote Bcl-2 regulation [295]. The authors observed that curcumin was capable to downregulate the Ets-1 gene and reduce Bcl-2 expression. For HEC-1-A cells, it was found DNA fragmentation induced by curcumin in a dose-dependent manner.

The in vivo effect of Curcumin on Bcl-2 and Bax expression was described using nude mice prostate cancer (PC3 cell line) [296]. Three groups were treated with different concentrations of this compound and showed an expressive reduction in tumor volume at all concentrations compared to control groups.

Huang and colleagues have shown the apoptotic effect of resveratrol in nasopharyngeal carcinoma cells. In their study, Bcl-2 was downregulated and Bax protein was upregulated. The expressive increase in the Bax/Bcl-2 ratio is responsible for the apoptosis due to the apoptotic properties of Bax. Besides that, it was also observed the release of cytochrome c due to the disruption of the mitochondrial membrane potential, and the activation of caspase-9 and -3. The last one responsible to cause DNA fragmentation and apoptosis [297].

Corroborating with previous results, Wang and co-workers have demonstrated in human leukemia cells the apoptotic effect of resveratrol and its ability to interfere in the regulation of proteins of Bcl-2 family. The ratio Bax/Bcl-2 increases, which induces the permeabilization of the outer mitochondrial membrane and the release of pro-apoptotic proteins. In their study, it was shown the decrease of cytochrome c level of the intermembrane space in the mitochondria and its increase in the cytosol. In addition, caspase-3 activity was increased as well [298].

Cholangiocarcinoma, human acute leukemia, liver and pancreatic cancer cell lines have demonstrated to be sensitive to resveratrol. In all four-cell lines, this polyphenol was able to induce apoptosis by reducing Bcl-2 levels and increase caspase-3 activity. Furthermore, in pancreatic cells was also demonstrated an up-regulation in Bax and downregulation in Bcx-xL and XIAP, and in liver cancer cells an increase in p53 expression protein was also detected [299,300,301].

#### 4.1.4. p53 Family

The TP53 gene is responsible for p53 protein codification, which is a transcription factor involved in cellular regulation, as well as, tumor suppression. Its effect occurs due to activation of repair proteins or induction of apoptosis, when cellular damages are irreversible [302,303,304]. This factor are present in both intrinsic and extrinsic pathways, and acts on the changes of mitochondrial membrane potential as cell sensitization to apoptosis [304].

According to He and co-workers [57], curcumin can ameliorate the general health state of patients with colorectal cancer through the increase of p53 expression in tumor cells. This study conducted with 126 patients, revealed that curcumin promotes an increase in weight body of the individuals when compared to control group (vehicle). After surgery, immunoblotting assay revealed that anti-apoptotic protein Bcl-2 was reduced and Bax was elevated. TNF-α level was also lower than control group, probably for p53 modulation. Thus, the authors have suggested that curcumin can be used in the treatment to ameliorate cachexia in these patients.

In breast cancer, it was demonstrated that resveratrol was not able to induce p53 protein expression, but expressively increased the phosphorylation in Ser15, resulting in a higher level of phospho-p53. When phosphorylated, p53 protein reduce its interaction with MDM2, an oncoprotein that regulates it negatively, what results in cell cycle arrest or apoptosis [305].

Notch-1 is a transmembrane receptor that mediates intracellular signalling involved in cell differentiation and cell survival [306]. In glioblastoma cells were demonstrated that Notch-1 activation and p-53 restoration by resveratrol was correlated. Glioblastoma cells were treated with a Notch-1 inhibitor (MRK-003) and resulted in a decrease of p53 restoration and significantly inhibition of p53 translocation to the nucleus, which indicates that Notch-1 activated is able to augment p53 expression and restore its function. In these cells, the activation of Notch-1-p53 signaling pathway indicates to be an initiating factor of apoptosis induced by resveratrol, with increased Bax expression and decreased Bcl-2 expression [307].

p73 is another transcription factor, belonging to p53 family, related to apoptosis and cancer progression. The p73 presents several functions in nervous system. Structurally, it is more complex than p53 because the conserved region in DNA-binding domain is also more complex [303]. p73 is responsible to perform the transcription of two isoform of proteins: TAp73 (related with tumor suppression and chemotherapy induced-apoptosis); and DNp73 (present in tumor cells and associated with chemoresistance) [308,309]. A research with p73 transfected Hep3B (p53-deficient) showed apoptosis induction when treated with curcumin at concentrations ranging from 40 to 80 µM. Western blot data have revealed an increase of TAp73 and reduction of DNp73 protein in the same concentrations necessary to induce apoptosis. MMP (mitochondrial membrane potential) were reduced and it was accompanied for cytochrome c release, cleavage of pro-caspase-9, pro-caspase-3, and pro-PARP [310].

#### 4.1.5. Extrinsic Pathway (Receptor-Mediated Pathway)

The extrinsic pathway is mediated by triggering cell surface death receptors of the tumor necrosis factor (TNF) receptor superfamily (TNF-R1, Fas/CD95, TRAIL-R1/DR4 and TRAIL-R2/DR5). After that, an adaptor, FADD (Fas-associated death domain protein), for example, binds to the receptor and a trimerized receptor-ligand complex (DISC—death-inducing signaling complex) is shaped. Thus, DISC recruits the initiator caspase-8, which is now activated [311]. In type I cells, caspase-8 activation is sufficient to apoptosis occurrence as a direct consequence, with activating downstream caspases such as caspase-3. In type II cells, the apoptosis is dependent on the amplification of death receptors via the mitochondrial pathway. The link between these two pathways occurs via Bid cleavage by caspase-8. The truncated bid interacts with Bax, promoting cytochrome c release and downstream events [312].

TRAIL (TNF-related apoptosis-inducing ligand) is the ligand of the death receptors DR4 and DR5. Some types of cells, like LNCaP (prostate cancer), are resistant to TRAIL-induced apoptosis. Shankar et al. have studied the resveratrol and curcumin ability to sensitize this prostate cancer cells to TRAIL. The results have demonstrated that these polyphenols were able to sensitize the cells to TRAIL, and they were also able to upregulate the TRAILs receptors, DR4 and DR5. Furthermore, the death receptor pathway was demonstrated to be involved in sensitization of TRAIL-resistant cells by resveratrol and curcumin [313,314].

An in vivo study with curcumin corroborates with the data above. LNCaP cells were xenografted in Balb nude mice and treatments with curcumin, TRAIL and curcumin + TRAIL was evaluated. Curcumin alone is able to induce apoptosis in tumor cells, while TRAIL is ineffective. When together, they are able to increase the cell death to values higher than curcumin alone, demonstrating that this natural product sensitize TRAIL-resistant cells [156].

In chondrosarcoma cells, curcumin was able to induce the cleavage of caspase-3, -7 and -8, but not -9, which indicates the activation of extrinsic pathway. Furthermore, it was also demonstrated an increase in Fas, FasL and DR5 expression by curcumin treatment, and transfection with siRNA of this components reduced apoptosis. p53 was also evaluated in this study, and it was shown to be able to participate of death receptor increased expression. Taken together, these results suggest that curcumin-induced cell death in chondrosarcoma cells occurs by extrinsic pathway [315].

In anaplastic large-cell lymphoma, resveratrol has induced apoptosis in a dose-dependent manner. In the same study, it was demonstrated that this phytoalexin was also able to induce the expression of the death receptor Fas/CD95 about twice folds when cells were treated with 25 µM of resveratrol for 48 h, indicating that extrinsic pathway may be a mechanism of this cellular apoptosis [316].

A link between intrinsic and extrinsic apoptotic pathway induced by resveratrol was demonstrated in multiple myeloma and T-cell leukemia cells. In the death receptor pathway, resveratrol induced the association of membrane rafts and Fas/CD95 and translocated DR4 and DR5 (TRAIL-receptors) to rafts. FADD, procaspase-8 and -10 were also translocated into rafts, as well as its actives forms. These data indicate that the constituents of DISC (FADD, Fas/CD95 and procaspase-8) are recruited into rafts, and this apoptotic complex in death receptor signaling is activated. Furthermore, Bid, which is a linker between Fas signaling and mitochondria was also translocated to raft. This data indicates a connection between intrinsic and extrinsic apoptotic pathway, which was demonstrated by blocking Fas/CD95 downstream signaling what prevented loss in membrane mitochondrial potential [317].

##### Endoplasmatic Reticulum (ER) Stress

Curcumin promotes apoptosis induction at a dose and time-dependent manner in human lung cancer cells. Besides the upregulation of the pro-apoptotic proteins Bax and Bad, an increased level of ROS accompanied for ER stress in these cells after treatment with curcumin was observed. These alterations conduce to MMP (mitochondrial membrane potential) modification and caspase-3 activation. The authors concluded that an activation of extrinsic pathway through increased FAS/CD95 expression promotes caspase-8 activation. This data was confirmed by using a caspase-8 inhibitor, which decreased the apoptosis in these cells [318].

#### 4.1.6. NF-κβ

The levels of NF-κβ are increased in pancreatic carcinoma cells. It was demonstrated that curcumin reduces this levels, promotes apoptosis and inhibits cellular proliferation. Reduction in the levels of I-κB kinase (IKK), NF-κβ, as well as, cyclooxygenase-2 (COX-2), prostaglandin E2 (PGE-2), and interleukin-8 (IL-8) were observed after treatment using curcumin [319].

Similar results were obtained using melanoma cells, where curcumin inhibited NF-kβ and IKK independently from B-Raf mutations or PI3K/Akt pathway. The authors did not found a direct correlation between IL-8 and NF-κβ for melanoma cells, and they hypothesized that IL-8 regulation could occur through AP-1 transcription factor [48].

In a different study using glioblastoma cells, curcumin was selective against cancer cells and promoted a reduction in NF-κβ and IKK leading to apoptosis [320].

Sun et al. have investigated the role of the inhibition of NF-κB in resveratrol-induced apoptosis in human multiple myeloma cells. When activated, p65 subunit of NF-κB is translocated to the nucleus, which lead the researches to evaluate its presence in the cytoplasm. As result, they found the vast majority of NF-κB in this compartment, where it could not function as transcription factor. Furthermore, the targets genes of NF-κB were also evaluated, and as expected, they were down regulated. Bcl-2, Bcl-xL, XIAP, c-IAP and VEGF are proteins resultant from the target genes activated by NF-κB [321].

Another example of the role of NF-κB in resveratrol-induced apoptosis was demonstrated in human breast cancer cells. EMSA experiments have shown a decrease in the p65(RelA)/p50 binding to the DNA at resveratrol levels that induces apoptosis. This result may be attributed to the lower level of NF-κB activated in nucleus due to the increase of the protein I-κB in the cytosol. These data were confirmed through the dose-dependent increased level of p65/(RelA) immunoprecipitated by an anti I-κB antibody. In this case, Bcl-2 was down regulated [322].

A study with multiple myeloma cells has demonstrated the ability of resveratrol to suppress the constitutively active IKK, which is necessary for NF-κB activation. Furthermore, resveratrol also inhibited the appearance of subunit p65 in the nucleus [323].

#### 4.1.7. PI3K, Akt/mTOR

Phosphotydilinositol-3 kinase (PI3K) is a lipid kinase family, which is activated by receptors with protein tyrosine kinase activity (RPTK). When RPTK is activated, PI3K associates with the receptor leading to the catalytic subunit activation and formation of the second messenger phosphatidylinositol-3,4,5-trisphosphate (PIP3). PIP3 recruits signaling proteins with pleckstrin homology (PH) domains to the membrane, including PDK1 and Akt. Akt activated has the ability to modulate the function of various substrates that are involved in cell survival, cell cycle progression and cellular growth [221].

Akt/PI3K is an important pathway for apoptosis regulation. In breast cancer cells, curcumin induced an Akt and glycogen synthase kinase 3b (GSK3B) phosphorylation. This kinase is involved in apoptosis process [324]. However, curiously in both cells: T-cell acute lymphoblastic leukemia (T-ALL) malignant cells and upper aero-digestive tract cancer cell; curcumin promotes the de-phosphorylation/inactivation of Akt, FOXO transcription factor and GSK3 [325,326].

FOXO transcription factors have been correlated with induction and cancer regulation. Pancreatic cancer cells treated with curcumin, presented an increased in FOXO1 (Forkhead box O1) expression, which is correlated with inhibition in phosphorylation/activation of PI3K and Akt [327].

mTOR, an Akt upstream modulator, was inhibited in vitro by curcumin using uterine leiomyosarcoma cells. Western Blot data revealed that curcumin has restrained p70S6 and S6 phosphorylations; both ribosomal proteins are downstream targets of mTOR. Interestingly, in the presence of a mTOR inhibitor (rapamycin), it was not observed apoptosis [328]. In vivo assay, using female nude mice, shows that curcumin decreases m-TOR and S6 phosphorylation leading to a reduction in tumor size [329].

In a time-dependent manner, resveratrol was able to reduce Akt phosphorylation, decrease the level of Akt protein and the phosphorylation of caspase-9, sequentially, in human breast cancer cells. Assuming that caspase-9 is a site for Akt and now it is activated, it indicates that this is one of the pathways for resveratrol-induced apoptosis [330].

Another pathway involving Akt activity and resveratrol-induced apoptosis was studied in human chronic myeloid leukemia cells. Hsp70, a heat shock protein, is responsible for helping the cell to maintain protein homeostasis and scape apoptosis and, in the cited cells, is overexpressed. The expression of Hsp genes is regulated by transcription factors of HSF (heat shock factor) family. In this study, resveratrol was able to decrease the phosphorylation of Akt, which is essential for its activity. GSK3B is a target of Akt and its phosphorylated form is inactive. Assuming that Akt is not able to phosphorylate GSK3B, then it is able to prevent HSF-1 to enter the nucleus and activate Hsp70 expression [331,332].

Studies have demonstrated that Akt is a direct regulator of miR21 expression [333]. PC-3M-MM2 cells exhibit a high level of phosphorylated Akt, which it is shown, in this study, to be decreased by resveratrol as well as miR-21 expression. To corroborate with this supposition, this androgen-independent human prostate carcinoma cells was treated with LY294002, a well-known inhibitor of Akt activity. The results demonstrated that the expression of miR-21 was also decreased, indicating that Akt may be a target for cancer treatment [334].

Dai et al. have studied in chondrosarcoma cells the ability of resveratrol to interfere in PI3K activity. By western blot analysis, it was demonstrated that the PI3K, Akt and AMPK levels decreased significantly in a concentration of resveratrol enough to cause apoptosis. This result suggest that the inhibition of PI3K pathway by resveratrol may be a molecular mechanism to suppress cancer cell proliferation [335].

#### 4.1.8. Telomerase

Telomerase is a reverse transcriptase, responsible to regulation of telomeric length of chromosomes, doing addition of repetitive sequences with guanine. This enzyme is expressed in proliferations cells, as germinal cells and cancer [336].

High levels of telomerase are found in tumor cells, and studies suggest this target as potential for anticancer drug development. In human leukemia cells and acute myeloblastic leukemia cells curcumin has inhibited telomerase activity, at dose and time-dependent manner. This activity is probably due to suppression of translocation of the catalytic subunit of telomerase (TERT—telomerase reverse transcriptase) from nucleus to cytosol. Curcumin induced apoptosis by increasing Bax and reducing Bcl-2, which promotes activation of caspase-3 and release of cytochrome c. The authors have suggested that a relationship between curcumin-induced apoptosis parameters and telomerase inhibition can exist [337,338].

Similar results were obtained using brain tumor cells. Khaw and collaborators identified that curcumin binds to cell surface and hen seeps into the cytoplasm in order to initiate the apoptotic cascade. TRAP assay and PCR revealed that curcumin inhibited telomerase activity through the inhibition in hTERT mRNA expression. This effect provokes a reduction of a telomere size. Moreover, caspase-3 and caspase-7 levels are increased [339].

A study carried out with MCF-7 cells has demonstrated the effect of resveratrol in telomerase activity. In a dose dependent manner, resveratrol was able to decrease the cellular viability and induce apoptosis. These events were related to resveratrol ability to down regulated TLMA, reduce the level of hTERT (catalytic subunit of human telomerase reverse transcriptase) of the nuclear compartment, where it is able to elongate the telomere and increase its levels in the cytoplasm, indicating that this phitoalexin is able to interfere in the process of translocation of this subunit to the nucleus [340].

In A431 epidermoid carcinoma cells, resveratrol was able to inhibit telomerase activity in a dose independent manner. Moreover, resveratrol was also able to decrease the expression of hTERT by inhibition of RNA transcription [341].

#### 4.1.9. JAK/STAT

STAT-3 (Signal transducer and activator of transcription 3) is a protein that has a dual role in normal cells, as cytoplasmic signaling proteins and as nuclear transcription factors that activates diverse genes. Among the genes regulated by STATs are the genes that control proliferation, apoptosis, angiogenesis and immune responses [342]. Simplistically, JAK2 is a tyrosine kinase responsible for the phosphorylation and activation of STAT-3, which is now able to enter into the nucleus and activate its target genes [343].

In human leukemia cells curcumin reduced the nuclear expression of STAT-3, 5a and 5b in dose and time-dependent manner. In addition, STAT-5a and 5b was followed by truncated isoforms formation, indicating that curcumin was able to induce the cleavage of STAT-5 into its dominant negative variants (lacking the STAT5 *C*-terminal region). However, it was not observed modifications in STAT-1 expression, only reduction in its transactivation. STAT-3, 5a and 5b phosphorylation was maintained and mRNA of Jak-2 was reduced as well as cyclin D1 and v-src gene expression [344].

Similar results were obtained in other researches with primary effusion lymphoma, Hodgkin’s lymphoma, cutaneous T-cell lymphoma and melanoma cells. These studies have found that curcumin reduces phosphorylation in Jak-2 or Jak-1 and STAT-3. These regulations provoke an apoptosis induction, reduction in Bcl-2, activation in caspase-3 and PARP cleavage [345,346,347,348].

In head and neck tumor cells, STAT-3 is overexpressed in comparison to others tumor cells. It was shown that resveratrol has inhibited the constitutive activation of STAT-3 and JAK2, the tyrosine kinase of the Janus family responsible for the STAT-3 phosphorylation. Beyond that, resveratrol inhibited STAT-3-DNA binding, because of the decreased phosphorylation level, which inhibits STAT-3 to translocate to the nucleus. Furthermore, resveratrol was also able to induce the expression of SOCS-1 (suppressor of cytokine signaling 1) protein and mRNA. SOCS-1 is a negative regulator of STAT-3 by inhibiting JAK2. STAT-3 is also known for its expression regulation of various genes products involved in anti-apoptosis (Bcl-2, Bcl-xL, survivin and others), which was found to be downregulated in resveratrol treatment [349].

In NK leukemia cells, resveratrol, in a time and dose-dependent manner, inhibited constitutively phosphorylation of STAT-3 and JAK2, which resulted in a decrease of downstream anti-apoptotic proteins MCL1, surviving and Bcl-10 [350].

In bladder and ovarian cancer cells, beyond the inhibition of STAT-3 expression and phosphorylation, it was demonstrated the reduction of STAT-3 into the nucleus. In consequence of this event, STAT-3 downstream anti-apoptotic products genes were suppressed [351,352].

#### 4.1.10. miRNA

miRNAs are portions of RNA that can not be transcript in proteins, and lately several works have established its role in many diseases, including cancer. Despite of this importance, until now is not known its exact function in many human diseases [353].

According to the literature, Bcl-2 is a target of miRNA15a and miRNA16 [354]. In human breast adenocarcinoma (MCF-7 cells), it was observed a downregulation in Bcl-2 and upregulation of mi-R15a and mi-R16 when exposed to different concentration of curcumin. In breast carcinoma cell lines, it was also found that curcumin was capable to upregulate these miRNA and the use of anti-miRNA15a and anti-miRNA16 promoted a renovation of Bcl-2 expression. Thus, curcumin can induce miR-15a and miR-16 expression and it can probably serve as potential gene therapy targets for Bcl-2-overexpressing tumors [355].

Curcumin increased miRNA16 in A549 human lung adenocarcinoma cell line, but promoted a significantly downregulation in miRNA186*. Authors observed that the use of an inhibitor for mRNA186*, not only reduce cellular proliferation but also promote apoptosis, indicating that miR-186* may play an oncogenic role in the development of lung cancer. Moreover, it was observed that modifications in miR-186* levels cause changes in caspase-10 levels. This enzyme appears to be increased in cell treated with curcumin [356].

Another study showed the relationship between curcumin and miRNA186* in treatment of multidrug-resistant cells of lung carcinoma (A549/DDP cells). These cells are sensitive to curcumin treatment, which can modify miRNA186* expression. The authors concluded that mRNA-186* can be a target for lung cancer susceptible to curcumin treatment [357].

In human glioma cells, resveratrol was able to inhibit the expression of the microRNA 21 (miR-21) that is found to be overexpressed in this type of cancer. Furthermore, it was studied the involvement of miR-21 and the resveratrol-induced apoptosis in these cells. It was found that the downregulation of miR-21 expression decreases the phosphorylation of I-kB and nuclear p65 protein levels, which leads to an inactivation of NF-κB signaling and, consequently, apoptosis [358].

Bcl-2 is a key regulator of apoptosis and it has been reported to be positive regulated by miR-21. To analyze if this is the mechanism involved in resveratrol-induced apoptosis in pancreatic cancer cells, Liu et al. have studied this purpose. Real-time PCR has demonstrated the ability of resveratrol to decreased the expression of miR-21, and western blot has demonstrated that Bcl-2 is downregulated by resveratrol, but it is restored by overexpression of miR-21. These results indicate that in pancreatic cancer cells the apoptosis induced by resveratrol is due to inhibiting miR-21 regulation of Bcl-2 expression [359].

A study realized by Zhou et al. in bladder cancer cells, resulted in the same data that Liu et al. demonstrating the ability of resveratrol to reduce miR-21 and Bcl-2. Furthermore, this study was able to indicate that Akt also participates of this process. It was demonstrated that resveratrol inhibits miR-21 expression, and as a consequence decreases Akt phosphorylation and Bcl-2 expression. The inhibition of Bcl-2 was counteracted by an Akt stimulator, demonstrating that in these cells, resveratrol is able to induce apoptosis by the regulation of Akt/Bcl-2 signaling pathway by inhibiting miR-21 expression [360].

### 4.2. Autophagy

This kind of cellular death are characterized for the formation of vesicles with cellular organelles (autophagosome), that promote an auto phagocytic process [361,362]. An important difference when compared to apoptosis, is that autophagy do not promote chromatin condensation and it is accompanied by massive autophagic vacuolization of the cytoplasm [362]. At cellular level the autophagic death can be considered as reversible process, once the stimuli is removed the cellular death process is interrupted [362].

Curcumin can induce autophagy in glioma cell lines, regulated by simultaneous inhibition of the Akt/mTOR/p70S6K pathway and stimulation of the ERK1/2 pathway. The last one regulates extracellular signalization, and when are activated promote autophagy. In vivo models using nude mice have revealed that curcumin reduced the tumor size by inducing autophagy. The mechanism seems to be related to LC3, an autophagosome-specific protein, that was increased in tumor treated for this polyphenol [363].

AMP is a kinase involved in metabolism of eukaryotic cells and its deregulation seems to be related with cancer process [364]. Similarly, in human adenocarcinoma cell line curcumin has promoted an autophagy process that was not observed in human normal lung cells. In this study, the authors observed an increased phosphorylation of AMP (AMPK) and acetylCoA carboxylase. The use of a si-RNA knockdown of a catalytic subunit of AMP kinase (AMPKα1) promotes a reduction in LC3-II, suggesting that this pathway is important to autophagy in these cell lines [365].

An in vitro and in vivo study with breast cancer stem-like cells has demonstrated the ability of resveratrol to decreased the cell viability in both systems. Thus, the cell death by autophagy was studied. It was demonstrated that resveratrol treatment increased the number of autophagossomes, upregulated the expression of LC3-II, Beclin1 and Atg 7, which are required for autophagossome formation, and GFP-LC3-II puncta formation assay demonstrated an increase in the percentage of cells with autophagossomes compared with control. It was also demonstrated that resveratrol induces autophagy, at least partially, via suppressing Wnt/βcatenin signaling pathway [366].

In melanoma cells, resveratrol treatment has induced a dose and time-dependent accumulation of LC3-II, significantly upregulation of Beclin-1 and induction of the formation of LC3 puncta, suggesting that resveratrol induces autophagy in these cells, and this event is regulated by ceramides, which regulates Akt/mTOR pathway. Interestingly results appeared when the conversion of LC3-I in LC3-II and Beclin-1 formation were inhibited. The cytotoxic effect of resveratrol increased as well as the apoptosis. It indicates that, in this case, autophagy acts as a resistance mechanism against apoptotic cell death, and inhibition of this event could be a novel strategy of treatment [367].

Others apoptotic targets have been studied for curcumin (Table 4) and resveratrol (Table 5).

## 5. Perspectives

The antitumoral properties of resveratrol and curcumin have been described in a number of studies using different types of cancers, including lung, breast, colon, leukemia, lymphoma, melanoma, multiple myeloma, neuroblastoma, osteosarcoma, ovarian, pancreatic, and prostate [107,108,277,278]. The majority of these studies have evaluated the anticancer properties of resveratrol or curcumin by itself (no-association) through in vitro or in vivo assays [408,409]. These studies conducted to hypothesis about the mechanism of action, whereby these polyphenols acted in the cell through down- or upregulation of important proteins, transcription factors and cytokines. Nevertheless, these polyphenols present non-specific action, considering the wide range of molecular targets that they can act. These non-specific activities are in fact, very different from the traditional chemotherapeutics that hit only one (or very few targets) in most of the cases [410]. This plurality of molecular targets associated to polyphenols have been generating divergent opinions in literature about the real contribution that such phytochemicals may have in anticancer therapy [37,145,410,411,412,413]. Nonetheless, there are a number of reviews in literature that highlight the cancer chemoprevention effect exerted by these polyphenols [414,415,416,417,418,419]. This chemopreventive effect has been associated to the anti-inflammatory properties of these phytochemicals, especially through the antioxidant activity [420,421,422,423].

Not only those targets discussed in this review, but also ability to complex with the DNA was described for both polyphenols. Using infrared spectroscopy, it was demonstrated that curcumin is able to interact with guanine, adenine and thymine, and the backbone PO_2_ in the DNA structure. It was also shown the ability of curcumin to complex the RNA molecule, which maintain its A-RNA conformation upon curcumin complexation [424,425].

Furthermore, there are a variety of studies involving these polyphenols in combination with approved anti-cancer drugs and its implication in anticancer combination therapy. These studies highlight the application of curcumin and resveratrol along with anticancer drugs aiming to improve the efficacy of the treatment. We highlighted in Table 6 some examples of polyphenols and anticancer drugs in combination regimens evaluated in vitro or in vivo.

The combinations of polyphenols (resveratrol and curcumin) within anticancer drugs have demonstrated in several cases a synergic effect and it seems to be a useful strategy to treat cancer.

Studies involving humans to test both polyphenols against cancer is being performed. Table 7 and Table 8 describe the current studies registered in US at different stages. It is possible to observe a high number of studies recruiting volunteers, which reveals the interest in both polyphenols by scientific community. Not only treatment against cancer but also chemoprevention and palliative care is being investigated (Table 7 and Table 8).

## 6. Conclusions

Curcumin and resveratrol are natural products with promising anticancer activity. Both compounds can act against proliferation, metastasis and cellular death through different mechanisms. Not only in vitro, but also in vivo data have demonstrated the potential of these polyphenols to treat and prevent cancer. In addition, the association of these polyphenols with current anticancer drugs has demonstrated synergic effect useful to improve the treatment. Different groups worldwide are conducting several clinical trials aiming to investigate the beneficial effects of curcumin and resveratrol in humans. Therefore, the use of resveratrol and curcumin seems to contribute to anticancer therapy.

## Figures and Tables

**Table 1:** Antiproliferative targets for curcumin.

Target	Effect	Cancer Type	Reference
GRP78	downregulation	Colon	[117]
EphA2	downregulation	Melanoma	[118]
SOCS1 & 3	upregulation	Leukemia	[119]
Nrf2	downregulation	Breast	[120]
miR-15a/16-1	downregulation	Leukemia	[121]
DLEC1	upregulation	Colon	[122]
Skp2	downregulation	Glioma	[123]

**Table 2:** Antiproliferative targets for resveratrol.

Target	Effect	Cancer Type	Reference
PKC	downregulation	gastric	[124]
eEF1A2	downregulation	ovarian	[125]
pro-IGFII	upregulation	breast	[126]
PTEN	upregulation	breast	[127]
MIC-1	upregulation	pancreas	[128]
6-PF1K	inhibition	breast	[129]
RNF20	activation	breast	[130]
Nox5	upregulation	lung	[131]
uH2B	downregulation	glioma	[132]

**Table 3:** Antimetastatic targets for resveratrol.

Target	Effect	Cancer Type	Reference
MTA-1/HDAC	downregulation	prostate	[257]
EGFR	downregulation	ovarian	[258]
MALAT-1	downregulation	colorectal	[259]
TGF-β1/Smads	downregulation	colorectal	[260]
α5β1 integrins/hyaluronic acid	downregulation/upregulation	ovarian	[261]
tensin	upregulation	erythroleukemia	[262]
TGF-β1	downregulation	lung	[263]
COX-2	downregulation	colon adenocarcinoma	[264]
interleukin-18	downregulation	hepatic melanoma	[265]

**Table 4:** Others apoptotic targets for curcumin.

Target	Effect	Cancer Type	Reference
AP-2γ	inhibition	testicular	[368]
MST1	activation	melanoma	[369]
Hexoquinase-2	downregulation	colorectal	[370]
Skp2/Her2	downregulation	breast	[371]
GADD45/153	upregulation	lung	[372]
Proteasome	inhibition	colon	[373]
Aurora A	downregulation	bladder	[374]
AMPK	activation	colon	[375]
Cdc27/APC3	inhibition	medulloblastoma	[376]
HDAC 4	inhibition	medulloblastoma	[377]
PKC	downregulation	liver	[378]
Sp1	inhibition	lung	[379]
Microtúbulo	inhibition	breast	[380]
Ras/ERK signaling	activation	gastric	[381]
Fatty acid synthase	inhibition	liver	[382]

**Table 5:** Others examples of apoptotic targets for resveratrol.

Target	Effect	Cancer Type	Reference
5-LOX	downregulation	mammary	[383]
COX 2	upregulation	ovarian	[384,385]
∆Np63	downregulation	nasopharyngeal	[386]
Hexoquinase 2	downregulation	hepatocellular	[387]
MTA1	downregulation	prostate	[388]
Specificity protein 1	inhibition	mesothelioma	[389]
GADD45α/Annexin A1	upregulation	leukemia	[390]
p21	upregulation	breast	[391]
ASPP1	upregulation	breast	[392]
TIGAR	downregulation	Lung/breast	[393]
Casein kinase (CK2)	downregulation	prostate	[394]
IRE1α/XBP1	upregulation	Multiple myeloma	[395]
Androgen receptor	downregulation	prostate	[396]
Caspase-6	upregulation	colon	[397]
CHOP	upregulation	colon	[398]
Cathepsin L/B	activation	Cervical/colorectal	[399,400]
ATF3	upregulation	colorectal	[401]
Fatty acid synthase	downregulation	breast	[402]
Hedgehog signaling	downregulation	pancreas	[403]
Tristetraprolin	activation	glioma	[404]
SphK1/S1P	downregulation	leukemia	[405]
Proteasome	activation	leukemia	[406]
Pentose phosphate and talin-FAK pathway	downregulation	colon	[407]

**Table 6:** Combination therapy of polyphenols and approved anti-cancer drugs.

Polyphenol	Drug	Cancer Type	Reference
curcumin	cisplatin	lung	[426]
curcumin	cisplatin	head and neck	[427]
curcumin	valproic acid	leukemia	[428]
curcumin	gemcitabine	pancreatic	[429]
curcumin	5-fluorouracil	breast	[430]
curcumin	5-fluorouracil	gastric	[431]
curcumin	5-fluorouracil + oxaliplatin	colon	[432]
curcumin	bevacizumab	liver	[433]
curcumin	imatinib	leukemia	[434]
curcumin	paclitaxel	brain	[435]
curcumin	oxaliplatin	colorectal	[436]
curcumin	temozolomide	glioblastoma	[437]
curcumin	gefitinib	lung	[438]
resveratrol	cisplatin	ovarian	[439]
resveratrol	cisplatin	colorectal	[440]
resveratrol	5-fluorouracil	colorectal	[441]
resveratrol	5-fluorouracil	melanoma	[442]
resveratrol	doxorubicin	breast	[443]
resveratrol	doxorubicin	leukemia	[444]
resveratrol	melphalan	breast	[445]
resveratrol	temozolomide	glioma	[446]
resveratrol	gemcitabine	pancreatic	[447]
resveratrol	paclitaxel	neuroblastoma	[448]
resveratrol	tamoxifen	breast	[449]
resveratrol	cyclophosphamide	breast	[450]

**Table 7:** Human studies using curcumin in cancer.

**Cancer Treatment**			
**Intervention**	**Study**	**Status**	**NCT Number**
Curcumin and 5-fluoracil (5-FU)	Curcumin in combination with 5-FU for colon cancer	Recruiting	NCT02724202 (Phase 0)
Curcumin and capecitabine	Curcumin, capecitabine and radiation therapy followed by surgery for rectal cancer	Ongoing, but not recruiting	NCT00745134 (Phase II)
Curcumin	Trial of curcumin in advanced-pancreatic cancer	Completed	NCT00094445 (Phase II)
Curcumin	Phase II study of curcumin versus placebo for chemotherapy-treated breast cancer patients undergoing radiotherapy	Recruiting	NCT01740323 (Phase II)
Avastin and Curcumin	Avastin/folfiri in combination with curcumin in colorectal cancer patients with metastasis	Recruiting	NCT02439385 (Phase II)
Gemcitabine and curcumin	Gemcitabine With Curcumin for Pancreatic Cancer	Completed	NCT00192842 (Phase II)
Curcumin	Effect of Curcumin in Treatment of Squamous Cervical Intraepithelial Neoplasias (CINs)	Recruiting	NCT02554344
Gemcitabine, curcumin and celecoxib	Phase III Trial of Gemcitabine, Curcumin and Celebrex in Patients with Metastatic Colon Cancer	Unknown	NCT00295035
Curcumin and Docetaxel	Multicenter Study Comparing Taxotere Plus Curcumin Versus Taxotere Plus Placebo Combination in First-line Treatment of Prostate Cancer Metastatic Castration Resistant (CURTAXEL) (CURTAXEL)	Ongoing, but not recruiting	NCT02095717 (Phase II)
Curcumin and Docetaxel	Docetaxel With or Without a Phytochemical in Treating Patients with Breast Cancer	Recruiting	NCT00852332 (Phase II)
Gemcitabine, curcumin and celebrex	Phase III trial of gemcitabine, curcumin and celebrex in patients with advance or inoperable pancreatic cancer	Unknown	NCT00486460 (Phase III)
Curcumin and cholecalciferol	Curcumin and cholecalciferol in treating patients with previously untreated stage 0-II Chronic lymphocytic leukemia or small lymphocytic lymphoma	Recruiting	NCT0210042 (Phase II)
Curcumin and bioperine	Pilot study of curcumin (diferuloylmethane derivative) with or without bioperine in patients with multiple myeloma	Completed	NCT00113841
Curcumin	Use of curcumin for treatment of intestinal adenomas in familial adenomatous polyposis (FAP)	Recruiting	NCT00927485
Anthocyanins and curcumin	Randomized window of opportunity trial of anthocyanin extract and phospholipid curcumin in subjects with colorectal adenoma	Recruiting	NCT0194866 (Phase II)
Curcumin and Ashwagandha extract	Pilot study of curcumin formulation and Ashwagandha extract in advanced osteosarcoma	Unknown	NCT00689195 (Phase I/II)
Curcumin	Turmeric effect on reduction of serum prolactin and related hormonal change and adenoma size in prolactinoma patients	Unknown	NCT0134429 (Phase I)
**Adverse Effects Management Induced by Chemotherapy**			
**Intervention**	**Study**	**Status**	**NCT Number**
Curcumin	Curcumin for the Prevention of Radiation-induced Dermatitis in Breast Cancer Patients	Completed	NCT01042938 (Phase II)
Curcumin	Radiosensitizing and Radioprotectve Effects of Curcumin in Prostate Cancer	Completed	NCT01917890
Curcumin and FOLFOX	Combining Curcumin With FOLFOX Chemotherapy in Patients with Inoperable Colorectal Cancer (CUFOX)	Ongoing, but no Recruiting	NCT01490996 (Phase I/II)
Curcumin	Nanocurcumin for Prostate Cancer Patients Undergoing Radiotherapy (RT)	Recruiting	NCT02724618 (Phase II)
Curcumin and Tirosine kinase inhibitors	An Open-label Prospective Cohort Trial of Curcumin Plus Tyrosine Kinase Inhibitors (TKI) for EGFR -Mutant Advanced NSCLC (CURCUMIN)	Recruiting	NCT02321293 (Phase I)
Curcumin	Prophylactic Topical Agents in Reducing Radiation-Induced Dermatitis in Patients with Non-inflammatory Breast Cancer or Breast Cancer in Situ (Curcumin-II)	Ongoing, but no recruiting	NCT02556632 (Phase II)
Curcumin	Effect of curcumin addition to standard treatment on tumor-induced inflammation in endometrial carcinoma	Recruiting	NCT02017353 (Phase II)
Curcumin	Curcumin for prevention of oral mucositis in children using chemotherapy	Completed	NCT00475683 (Phase III)
Curcumin	Oral curcumin for radiation dermatitis in breast cancer patients	Completed	NCT01246973 (Phase II/III)
**Chemoprevention**			
**Intervention**	**Study**	**Status**	**NCT Number**
Curcumin	Curcumin in Treating Patients with Familial Adenomatous Polyposis	Ongoing, but not recruiting	NCT00641147 (Phase II)
Curcumin	Curcumin in Preventing Gastric Cancer in Patients with Chronic Atrophic Gastritis or Gastric Intestinal Metaplasia	Not yet recruiting	NCT02782949 (Phase II)
Curcumin	Sulindac and plant compounds in preventing colon cancer	Completed	NCT00003365
Curcumin	Curcumin for the chemoprevention of colorectal cancer	Completed	NCT00118989 (Phase II)
Curcumin and sulindac	The effects of curcuminoids on aberrant crypt foci in the human colon	Unknown	NCT00176618
Curcumin	Randomized trial of adjuvant curcumin after prostatectomy	Recruiting	NCT02064673

**Table 8:** Human studies using resveratrol in cancer.

**Cancer Treatment**			
**Intervention**	**Study**	**Status**	**NCT Number**
Resveratrol	Resveratrol for patients with colon cancer	Completed	NCT00256334 (Phase I)
Resveratrol	Resveratrol in treating patients with colorectal cancer that can be removed by surgery	Completed	NCT00433576 (Phase I)
Resveratrol	A biological study of resveratrol’s effects on notch-1 signaling in subjects with low grade gastrointestinal tumors	Ongoing, not recruiting	NCT01476592
Resveratrol and others	Dietary intervention in follicular lymphoma (KLYMF)	Unknown	NCT00455416 (Phase II)
**Adverse Effects Management Induced by Chemotherapy**			
**Intervention**	**Study**	**Status**	**NCT Number**
SRT501 (new formulation of resveratrol)	A clinical study to assess the safety, pharmacokinetics, and pharmacodynamics of SRT501 in subjects with colorectal cancer and hepatic metastases	Completed	NCT00920803 (Phase I)
**Chemoprevention**			
**Intervention**	**Study**	**Status**	**NCT Number**
Resveratrol	UMCC 2003-064 Resveratrol in Preventing Cancer in Healthy Participants (IRB 2004-535)	Completed	NCT00098969 (Phase I)

## References

[1] Cimino S., Sortino G., Favilla V., Castelli T., Madonia M., Sansalone S., Russo G.I., Morgia G. (2012). Polyphenols: Key issues involved in chemoprevention of prostate cancer. Oxid. Med. Cell. Longev..

[2] Rodríguez M.L., Estrela J.M., Ortega Á.L. (2013). Carcinogenesis & mutagenesis natural polyphenols and apoptosis Induction in cancer therapy. J. Carcinog. Mutagen..

[3] Esatbeyoglu T., Huebbe P., Ernst I.M.A., Chin D., Wagner A.E., Rimbach G. (2012). Curcumin—From molecule to biological function. Angew. Chem. Int. Ed..

[4] Hatcher H., Planalp R., Cho J., Torti F.M., Torti S.V. (2008). Curcumin: From ancient medicine to current clinical trials. Cell. Mol. Life Sci..

[5] Kunnumakkara A.B., Anand P., Aggarwal B.B. (2008). Curcumin inhibits proliferation, invasion, angiogenesis and metastasis of different cancers through interaction with multiple cell signaling proteins. Cancer Lett..

[6] Leischner C., Burkard M., Pfeiffer M.M., Lauer U.M., Busch C., Venturelli S. (2016). Nutritional immunology: Function of natural killer cells and their modulation by resveratrol for cancer prevention and treatment. Nutr. J..

[7] Chakraborty S., Kumar A., Butt N.A., Zhang L., Williams R., Rimando A.M., Biswas P.K., Levenson A.S. (2016). Molecular insight into the differential anti-androgenic activity of resveratrol and its natural analogs: In silico approach to understand biological actions. Mol. BioSyst..

[8] Das J., Ramani R., Suraju M.O. (2016). Polyphenol compounds and PKC signaling. Biochim. Biophys. Acta Gen. Subj..

[9] Walle T. (2011). Bioavailability of resveratrol. Ann. N. Y. Acad. Sci..

[10] Anand P., Kunnumakkara A.B., Newman R.A., Aggarwal B.B. (2007). Bioavailability of curcumin: Problems and promises. Mol. Pharm..

[11] Jäger R., Lowery R.P., Calvanese A.V., Joy J.M., Purpura M., Wilson J.M. (2014). Comparative absorption of curcumin formulations. Nutr. J..

[12] Zebib B., Mouloungui Z., Noirot V. (2010). Stabilization of curcumin by complexation with divalent cations in glycerol/water system. Bioinorg. Chem. Appl..

[13] Shoba G., Joy D., Joseph T., Majeed M., Rajendran R., Srinivas P.S. (1998). Influence of piperine on the pharmacokinetics of curcumin in animals and human volunteers. Planta Med..

[14] Suresh D., Srinivasan K. (2010). Tissue distribution & elimination of capsaicin, piperine & curcumin following oral intake in rats. Indian J. Med. Res..

[15] Xie X., Tao Q., Zou Y., Zhang F., Guo M., Wang Y., Wang H., Zhou Q., Yu S. (2011). PLGA nanoparticles improve the oral bioavailability of curcumin in rats: Characterizations and mechanisms. J. Agric. Food Chem..

[16] Tsai Y.-M., Jan W.-C., Chien C.-F., Lee W.-C., Lin L.-C., Tsai T.-H. (2011). Optimised nano-formulation on the bioavailability of hydrophobic polyphenol, curcumin, in freely-moving rats. Food Chem..

[17] Shaikh J., Ankola D.D., Beniwal V., Singh D., Kumar M.N.V.R. (2009). Nanoparticle encapsulation improves oral bioavailability of curcumin by at least 9-fold when compared to curcumin administered with piperine as absorption enhancer. Eur. J. Pharm. Sci..

[18] Yallapu M.M., Jaggi M., Chauhan S.C. (2010). Poly(β-cyclodextrin)/curcumin self-assembly: A novel approach to improve curcumin delivery and its therapeutic efficacy in prostate cancer cells. Macromol. Biosci..

[19] Sasaki H., Sunagawa Y., Takahashi K., Imaizumi A., Fukuda H., Hashimoto T., Wada H., Katanasaka Y., Kakeya H., Fujita M. (2011). Innovative preparation of curcumin for improved oral bioavailability. Biol. Pharm. Bull..

[20] Prasad S., Tyagi A.K., Aggarwal B.B. (2014). Recent developments in delivery, bioavailability, absorption and metabolism of curcumin: The golden pigment from golden spice. Cancer Res. Treat..

[21] Cuomo J., Appendino G., Dern A.S., Schneider E., Mckinnon T.P., Brown M.J., Togni S., Dixon B.M. (2011). Comparative absorption of a standardized curcuminoid mixture and its lecithin formulation. J. Nat. Prod..

[22] Imaizumi A. (2015). Highly bioavailable curcumin (Theracurmin): Its development and clinical application. PharmaNutrition.

[23] Sunagawa Y., Katanasaka Y., Hasegawa K., Morimoto T. (2015). Clinical applications of curcumin. PharmaNutrition.

[24] Smoliga J.M., Blanchard O. (2014). Enhancing the delivery of resveratrol in humans: If low bioavailability is the problem, what is the solution?. Molecules.

[25] Wang S., Su R., Nie S., Sun M., Zhang J., Wu D., Moustaid-Moussa N. (2014). Application of nanotechnology in improving bioavailability and bioactivity of diet-derived phytochemicals. J. Nutr. Biochem..

[26] Pageni R., Sahni J.K., Ali J., Sharma S., Baboota S. (2014). Resveratrol: Review on therapeutic potential and recent advances in drug delivery. Expert Opin. Drug Deliv..

[27] Sessa M., Tsao R., Liu R., Ferrari G., Donsì F. (2011). Evaluation of the stability and antioxidant activity of nanoencapsulated resveratrol during in vitro digestion. J. Agric. Food Chem..

[28] Ansari K.A., Vavia P.R., Trotta F., Cavalli R. (2011). Cyclodextrin-based nanosponges for delivery of resveratrol: In vitro characterisation, stability, cytotoxicity and permeation study. AAPS Pharm. Sci. Tech..

[29] Liang L., Liu X., Wang Q., Cheng S., Zhang S., Zhang M. (2013). Pharmacokinetics, tissue distribution and excretion study of resveratrol and its prodrug 3,5,4’-tri-*O*-acetylresveratrol in rats. Phytomedicine.

[30] Johnson J.J., Nihal M., Siddiqui I.A., Scarlett C.O., Bailey H.H., Mukhtar H., Ahmad N. (2011). Enhancing the bioavailability of resveratrol by combining it with piperine. Mol. Nutr. Food Res..

[31] Cottart C.-H., Nivet-Antoine V., Beaudeux J.-L. (2014). Review of recent data on the metabolism, biologicaleffects, and toxicity of resveratrol in humans. Mol. Nutr. Food Res..

[32] Cottart C.-H., Nivet-Antoine V., Laguillier-Morizot C., Beaudeux J.-L. (2010). Resveratrol bioavailability and toxicity in humans. Mol. Nutr. Food Res..

[33] Mukherjee S., Dudley J.I., Das D.K. (2010). Dose-dependency of resveratrol in providing health benefits. Dose Response.

[34] Lao C.D., Ruffin M.T., Normolle D., Heath D.D., Murray S.I., Bailey J.M., Boggs M.E., Crowell J., Rock C.L., Brenner D.E. (2006). Dose escalation of a curcuminoid formulation. BMC Complement. Altern. Med..

[35] Dutra L.A., de Melo T.R.F. (2016). The paradigma of the interference in assays for natural products. Biochem. Pharmacol..

[36] Dos Santos J.L., Chin C.M. (2015). Pan-assay interference compounds (PAINS): Warning signs in biochemical-pharmacological evaluations. Biochem. Pharmacol..

[37] Ingólfsson H.I., Thakur P., Herold K.F., Hobart E.A., Ramsey N.B., Periole X., de Jong D.H., Zwama M., Yilmaz D., Hall K. (2014). Phytochemicals perturb membranes and promiscuously alter protein function. ACS Chem. Biol..

[38] Shishodia S. (2013). Molecular mechanisms of curcumin action: Gene expression. Biofactors.

[39] Coussens L.M., Werb Z. (2002). Inflammation and cancer. Nature.

[40] Mantovani A., Allavena P., Sica A., Balkwill F. (2008). Cancer-related inflammation. Nature.

[41] Karin M. (2006). Nuclear factor-kappaB in cancer development and progression. Nature.

[42] Balkwill F., Mantovani A. (2001). Inflammation and cancer: Back to virchow?. Lancet.

[43] Jobin C., Bradham C.A., Russo M.P., Juma B., Narula A.S., Brenner D.A., Sartor R.B. (1999). Curcumin blocks cytokine-mediated NF-κB activation and proinflammatory gene expression by inhibiting inhibitory factor I-κB kinase activity. J. Immunol..

[44] Aggarwal B.B. (2004). Nuclear factor-kappaB: The enemy within. Cancer Cell.

[45] Pahl H.L. (1999). Activators and target genes of Rel/NF-kappaB transcription factors. Oncogene.

[46] Baldwin A.S. (2001). Series introduction: The transcription factor NF-kappaB and human disease. J. Clin. Investig..

[47] Liu S., Wang Z., Hu Z., Zeng X., Li Y., Su Y., Zhang C., Ye Z. (2011). Anti-tumor activity of curcumin against androgen-independent prostate cancer cells via inhibition of NF-κB and AP-1 pathway in vitro. J. Huazhong Univ. Sci. Technol. Med. Sci..

[48] Siwak D.R., Shishodia S., Aggarwal B.B., Kurzrock R. (2005). Curcumin-induced antiproliferative and proapoptotic effects in melanoma cells are associated with suppression of IkappaB kinase and nuclear factor kappaB activity and are independent of the B-Raf/mitogen-activated/extracellular signal-regulated protein ki. Cancer.

[49] Hu L., Xia L., Zhou H., Wu B., Mu Y., Wu Y., Yan J. (2013). TF/FVIIa/PAR2 promotes cell proliferation and migration via PKCα and ERK-dependent c-Jun/AP-1 pathway in colon cancer cell line SW620. Tumor Biol..

[50] Balasubramanian S., Eckert R.L. (2007). Curcumin suppresses AP1 transcription factor-dependent differentiation and activates apoptosis in human epidermal keratinocytes. J. Biol. Chem..

[51] Ruocco K.M., Goncharova E.I., Young M.R., Colburn N.H., McMahon J.B., Henrich C.J. (2006). A high-throughput cell-based assay to identify specific inhibitors of transcription factor AP-1. J. Biomol. Screen..

[52] Chen Q., Jiao D., Wang L., Wang L., Hu H., Song J., Yan J., Wu L., Shi J. (2015). Curcumin inhibits proliferation-migration of NSCLC by steering crosstalk between a Wnt signaling pathway and an adherens junction via EGR-1. Mol. Biosyst..

[53] Byeong H.C., Chang G.K., Bae Y.S., Lim Y., Young H.L., Soon Y.S. (2008). p21Waf1/Cip1 expression by curcumin in U-87MG human glioma cells: Role of early growth response-1 expression. Cancer Res..

[54] Chen A., Xu J., Johnson A. (2006). Curcumin inhibits human colon cancer cell growth by suppressing gene expression of epidermal growth factor receptor through reducing the activity of the transcription factor Egr-1. Oncogene.

[55] Hong J.H., Lee G., Choi H.Y. (2015). Effect of curcumin on the interaction between androgen receptor and Wnt/β-catenin in LNCaP xenografts. Korean J. Urol..

[56] Choi H.Y., Lim J.E., Hong J.H. (2010). Curcumin interrupts the interaction between the androgen receptor and Wnt/beta-catenin signaling pathway in LNCaP prostate cancer cells. Prostate Cancer Prostatic Dis..

[57] He M., Li Y., Zhang L., Li L., Shen Y., Lin L., Zheng W., Chen L., Bian X., Ng H.K., Tang L. (2014). Curcumin suppresses cell proliferation through inhibition of the Wnt/β-catenin signaling pathway in medulloblastoma. Oncol. Rep..

[58] Alonso A., Sasin J., Bottini N., Friedberg I., Friedberg I., Osterman A., Godzik A., Hunter T., Dixon J., Mustelin T. (2004). Protein tyrosine phosphatases in the human genome. Cell.

[59] Hunter T. (1987). A thousand and one protein kinases. Cell.

[60] Manning G., Whyte D.B., Martinez R., Hunter T., Sudarsanam S. (2002). The protein kinase complement of the human genome. Science.

[61] Roskoski R. (2015). A historical overview of protein kinases and their targeted small molecule inhibitors. Pharmacol. Res..

[62] Cohen P. (2001). The role of protein phosphorylation in human health and disease. Eur. J. Biochem..

[63] Blume-Jensen P., Hunter T. (2001). Oncogenic kinase signalling. Nature.

[64] Lahiry P., Torkamani A., Schork N.J., Hegele R. (2010). A kinase mutations in human disease: Interpreting genotype-phenotype relationships. Nat. Rev. Genet..

[65] Fabbro D., Cowan-Jacob S.W., Moebitz H. (2015). Ten things you should know about protein kinases: IUPHAR Review 14. Br. J. Pharmacol..

[66] Yang M., Huang C.-Z. (2015). Mitogen-activated protein kinase signaling pathway and invasion and metastasis of gastric cancer. World J. Gastroenterol..

[67] Yesilkanal A.E., Rosner M.R. (2014). Raf kinase inhibitory protein (RKIP) as a metastasis suppressor: Regulation of signaling networks in cancer. Crit. Rev. Oncog..

[68] Almhanna K., Strosberg J., Malafa M. (2011). Targeting AKT protein kinase in gastric cancer. Anticancer Res..

[69] Zhou H., Huang S. (2011). Role of mTOR signaling in tumor cell motility, invasion and metastasis. Curr. Protein Pept. Sci..

[70] Rattanasinchai C., Gallo K. (2016). MLK3 signaling in cancer invasion. Cancers.

[71] Varkaris A., Katsiampoura A.D., Araujo J.C., Gallick G.E., Corn P.G. (2014). Src signaling pathways in prostate cancer. Cancer Metastasis Rev..

[72] Durand N., Borges S., Storz P. (2015). Functional and therapeutic significance of protein kinase D enzymes in invasive breast cancer. Cell. Mol. Life Sci..

[73] Igea A., Nebreda A.R. (2015). The stress kinase p38α as a target for cancer therapy. Cancer Res..

[74] Li N., Huang D., Lu N., Luo L. (2015). Role of the LKB1/AMPK pathway in tumor invasion and metastasis of cancer cells (Review). Oncol. Rep..

[75] Starok M., Preira P., Vayssade M., Haupt K., Salomé L., Rossi C. (2015). EGFR inhibition by curcumin in cancer cells: A dual mode of action. Biomacromolecules.

[76] Chadalapaka G., Jutooru I., Burghardt R., Safe S. (2010). Drugs that target specificity proteins downregulate epidermal growth factor receptor in bladder cancer cells. Mol. Cancer Res..

[77] Amani V., Prince E.W., Alimova I., Balakrishnan I., Birks D., Donson A.M., Harris P., Levy J.M.M., Handler M., Foreman N.K. (2016). Polo-like Kinase 1 as a potential therapeutic target in Diffuse Intrinsic Pontine Glioma. BMC Cancer.

[78] Van Erk M.J., Teuling E., Staal Y.C., Huybers S., Van Bladeren P.J., Aarts J.M., Van Ommen B. (2004). Time- and dose-dependent effects of curcumin on gene expression in human colon cancer cells. J. Carcinog..

[79] Mosieniak G., Sliwinska M.A., Przybylska D., Grabowska W., Sunderland P., Bielak-Zmijewska A., Sikora E. (2016). Curcumin-treated cancer cells show mitotic disturbances leading to growth arrest and induction of senescence phenotype. Int. J. Biochem. Cell Biol..

[80] Downward J. (2004). PI 3-kinase, Akt and cell survival. Semin. Cell Dev. Biol..

[81] Minet E., Michel G., Mottet D., Raes M., Michiels C. (2001). Transduction pathways involved in hypoxia-inducible factor-1 phosphorylation and activation. Free Radic. Biol. Med..

[82] Zhang Q., Tang X., Lu Q.Y., Zhang Z.F., Brown J., Le A.D. (2005). Resveratrol inhibits hypoxia-induced accumulation of hypoxia-inducible factor-1α and VEGF expression in human tongue squamous cell carcinoma and hepatoma cells. Mol. Cancer Ther..

[83] Faber A.C., Dufort F.J., Blair D., Wagner D., Roberts M.F., Chiles T.C. (2006). Inhibition of phosphatidylinositol 3-kinase-mediated glucose metabolism coincides with resveratrol-induced cell cycle arrest in human diffuse large B-cell lymphomas. Biochem. Pharmacol..

[84] Benitez D.A., Pozo-Guisado E., Clementi M., Castellón E.A., Fernandez-Salguero P.M. (2007). Non-genomic action of resveratrol on androgen and oestrogen receptors in prostate cancer: Modulation of the phosphoinositide 3-kinase pathway. Br. J. Cancer.

[85] Benitez D.A., Hermoso M.A., Pozo-Guisado E., Fernández-Salguero P.M., Castellón E.A. (2009). Regulation of cell survival by resveratrol involves inhibition of NFκβ-regulated gene expression in prostate cancer cells. Prostate.

[86] Hsieh T.C., Wong C., Bennett D.J., Wu J.M. (2011). Regulation of p53 and cell proliferation by resveratrol and its derivatives in breast cancer cells: An in silico and biochemical approach targeting integrin αvβ3. Int. J. Cancer.

[87] De Amicis F., Giordano F., Vivacqua A., Pellegrino M., Panno M.L., Tramontano D., Fuqua S.A.W., Ando S. (2011). Resveratrol, through NF-Y/p53/Sin3/HDAC1 complex phosphorylation, inhibits estrogen receptor gene expression via p38MAPK/CK2 signaling in human breast cancer cells. FASEB J..

[88] Abusnina A., Keravis T., Yougbaré I., Bronner C., Lugnier C. (2011). Anti-proliferative effect of curcumin on melanoma cells is mediated by PDE1A inhibition that regulates the epigenetic integrator UHRF1. Mol. Nutr. Food Res..

[89] Abusnina A., Keravis T., Zhou Q., Justiniano H., Lobstein A., Lugnier C. (2015). Tumour growth inhibition and anti-angiogenic effects using curcumin correspond to combined PDE2 and PDE4 inhibition. Thromb. Haemost..

[90] Weis S.M., Cheresh D.A. (2011). Tumor angiogenesis: Molecular pathways and therapeutic targets. Nat. Med..

[91] Zhao Y., Adjei A.A. (2015). Targeting angiogenesis in cancer therapy: Moving beyond vascular endothelial growth factor. Oncologist.

[92] Shojaei F. (2012). Anti-angiogenesis therapy in cancer: Current challenges and future perspectives. Cancer Lett..

[93] Ellis L.M., Hicklin D.J. (2008). VEGF-targeted therapy: Mechanisms of anti-tumour activity. Nat. Rev. Cancer.

[94] Ferrara N., Gerber H.P., LeCouter J. (2003). The biology of VEGF and its receptors. Nat. Med..

[95] Bae M.K., Kim S.H., Jeong J.W., Lee Y.M., Kim H.S., Kim S.R., Yun I., Bae S.K., Kim K.W. (2006). Curcumin inhibits hypoxia-induced angiogenesis via down-regulation of HIF-1. Oncol. Rep..

[96] Shan B., Schaaf C., Schmidt A., Lucia K., Buchfelder M., Losa M., Kuhlen D., Kreutzer J., Perone M.J., Arzt E. (2012). Curcumin suppresses HIF1A synthesis and VEGFA release in pituitary adenomas. J. Endocrinol..

[97] Fu Z., Chen X., Guan S., Yan Y., Lin H., Hua Z.-C. (2015). Curcumin inhibits angiogenesis and improves defective hematopoiesis induced by tumor-derived VEGF in tumor model through modulating VEGF-VEGFR2 signaling pathway. Oncotarget.

[98] Dann J.M., Sykes P.H., Mason D.R., Evans J.J. (2009). Regulation of vascular endothelial growth factor in endometrial tumour cells by resveratrol and EGCG. Gynecol. Oncol..

[99] Hu Y., Sun C., Huang J., Hong L., Zhang L., Chu Z. (2007). Antimyeloma effects of resveratrol through inhibition of angiogenesis. Chin. Med. J..

[100] Liu Z., Li Y., Yang R. (2012). Effects of resveratrol on vascular endothelial growth factor expression in osteosarcoma cells and cell proliferation. Oncol. Lett..

[101] Yang R., Zhang H., Zhu L. (2011). Inhibitory effect of resveratrol on the expression of the VEGF gene and proliferation in renal cancer cells. Mol. Med. Rep..

[102] Trapp V., Parmakhtiar B., Papazian V., Willmott L., Fruehauf J.P. (2010). Anti-angiogenic effects of resveratrol mediated by decreased VEGF and increased TSP1 expression in melanoma-endothelial cell co-culture. Angiogenesis.

[103] Mousa S.S., Mousa S.S., Mousa S.A. (2005). Effect of resveratrol on angiogenesis and platelet/fibrin-accelerated tumor growth in the chick chorioallantoic membrane model. Nutr. Cancer.

[104] Ravindran J., Prasad S., Aggarwal B.B. (2009). Curcumin and cancer cells: How many ways can curry kill tumor cells selectively?. AAPS J..

[105] Yang C.L., Liu Y.Y., Ma Y.G., Xue Y.X., Liu D.G., Ren Y., Liu X.B., Li Y., Li Z. (2012). Curcumin blocks small cell lung cancer cells migration, invasion, angiogenesis, cell cycle and neoplasia through janus kinase-STAT3 signalling pathway. PLoS ONE.

[106] Lim T.-G., Lee S.-Y., Huang Z., Lim D.Y., Chen H., Jung S.K., Bode A.M., Lee K.W., Dong Z. (2014). Curcumin suppresses proliferation of colon cancer cells by targeting CDK2. Cancer Prev. Res..

[107] Hudson T.S., Hartle D.K., Hursting S.D., Nunez N.P., Wang T.T.Y., Young H.A., Arany P., Green J.E. (2007). Inhibition of prostate cancer growth by muscadine grape skin extract and resveratrol through distinct mechanisms. Cancer Res..

[108] Wang C., Hu Z., Chu M., Wang Z., Zhang W., Wang L., Li C., Wang J. (2012). Resveratrol inhibited GH3 cell growth and decreased prolactin level via estrogen receptors. Clin. Neurol. Neurosurg..

[109] Kim A.L., Zhu Y., Zhu H., Han L., Kopelovich L., Bickers D.R., Athar M. (2006). Resveratrol inhibits proliferation of human epidermoid carcinoma A431 cells by modulating MEK1 and AP-1 signalling pathways. Exp. Dermatol..

[110] Yuan L., Zhang Y., Xia J., Liu B., Zhang Q., Liu J., Luo L., Peng Z., Song Z., Zhu R. (2015). Resveratrol induces cell cycle arrest via a p53-independent pathway in A549 cells. Mol. Med. Rep..

[111] Zhou R., Fukui M., Choi H.J., Zhu B.T. (2009). Induction of a reversible, non-cytotoxic S-phase delay by resveratrol: Implications for a mechanism of lifespan prolongation and cancer protection. Br. J. Pharmacol..

[112] Yu X.-D., Yang J., Zhang W.-L., Liu D.-X. (2016). Resveratrol inhibits oral squamous cell carcinoma through induction of apoptosis and G2/M phase cell cycle arrest. Tumor Biol..

[113] Carafa V., Nebbioso A., Altucci L. (2012). Sirtuins and disease: The road ahead. Front. Pharmacol..

[114] Yang Q., Wang B., Zang W., Wang X., Liu Z., Li W., Jia J. (2013). Resveratrol inhibits the growth of gastric cancer by inducing G1 phase arrest and senescence in a Sirt1-dependent manner. PLoS ONE.

[115] Lin J.-N., Lin V.C.-H., Rau K.-M., Shieh P.-C., Kuo D.-H., Shieh J.-C., Chen W.-J., Tsai S.-C., Way T.-D. (2010). Resveratrol modulates tumor cell proliferation and protein translation via SIRT1-dependent AMPK activation. J. Agric. Food Chem..

[116] Li Y., Zhu W., Li J., Liu M., Wei M. (2013). Resveratrol suppresses the STAT3 signaling pathway and inhibits proliferation of high glucose-exposed HepG2 cells partly through SIRT1. Oncol. Rep..

[117] Chang Y.J., Huang C.Y., Hung C.S., Chen W.Y., Wei P.L. (2015). GRP78 mediates the therapeutic efficacy of curcumin on colon cancer. Tumor Biol..

[118] Chen L.X., He Y.J., Zhao S.Z., Wu J.G., Wang J.T., Zhu L.M., Lin T.T., Sun B.C., Li X.R. (2011). Inhibition of tumor growth and vasculogenic mimicry by cucumin through downregulation of the EphA2/PI3K/MMP pathway in a murine choroidal melanoma model. Cancer Biol. Ther..

[119] Chen C.Q., Yu K., Yan Q.X., Xing C.Y., Chen Y., Yan Z., Shi Y.F., Zhao K.W., Gao S.M. (2013). Pure curcumin increases the expression of SOCS_1_ and SOCS_3_ in myeloproliferative neoplasms through suppressing class I histone deacetylases. Carcinogenesis.

[120] Chen B., Zhang Y., Wang Y., Rao J., Jiang X., Xu Z. (2014). Curcumin inhibits proliferation of breast cancer cells through Nrf2-mediated down-regulation of Fen1 expression. J. Steroid Biochem. Mol. Biol..

[121] Gao S., Yang J., Chen C., Chen J., Ye L., Wang L., Wu J., Xing C., Yu K. (2012). Pure curcumin decreases the expression of WT1 by upregulation of miR-15a and miR-16-1 in leukemic cells. J. Exp. Clin. Cancer Res..

[122] Guo Y., Shu L., Zhang C., Su Z.-Y., Kong A.-N.T. (2015). Curcumin inhibits anchorage-independent growth of HT29 human colon cancer cells by targeting epigenetic restoration of the tumor suppressor gene DLEC1. Biochem. Pharmacol..

[123] Wang L., Ye X., Cai X., Su J., Ma R., Yin X., Zhou X., Li H., Wang Z. (2015). Curcumin suppresses cell growth and invasion and induces apoptosis by down-regulation of Skp2 pathway in glioma cells. Oncotarget.

[124] Atten M.J., Godoy-romero E., Attar B.M., Milson T., Zopel M., Holian O. (2005). Resveratrol regulates cellular PKC α and δ to inhibit growth and induce apoptosis in gastric cancer cells. Investig. New Drugs.

[125] Lee M.-H., Choi B.Y., Kundu J.K., Shin Y.K., Na H.-K., Surh Y.-J. (2009). Resveratrol suppresses growth of human ovarian cancer cells in culture and in a murine xenograft model: Eukaryotic elongation factor 1A2 as a potential target. Cancer Res..

[126] Vyas S., Asmerom Y., De León D.D. (2005). Resveratrol regulates insulin-like growth factor-II in breast cancer cells. Endocrinology.

[127] Waite K.A., Sinden M.R., Eng C. (2005). Phytoestrogen exposure elevates PTEN levels. Hum. Mol. Genet..

[128] Golkar L., Ding X.Z., Ujiki M.B., Salabat M.R., Kelly D.L., Scholtens D., Fought A.J., Bentrem D.J., Talamonti M.S., Bell R.H., Adrian T.E. (2007). Resveratrol inhibits pancreatic cancer cell proliferation through transcriptional induction of Macrophage Inhibitory Cytokine-1. J. Surg. Res..

[129] Gomez L.S., Zancan P., Marcondes M.C., Ramos-Santos L., Meyer-Fernandes J.R., Sola-Penna M., Da Silva D. (2013). Resveratrol decreases breast cancer cell viability and glucose metabolism by inhibiting 6-phosphofructo-1-kinase. Biochimie.

[130] Lin C.Y., Hsiao W.C., Wright D.E., Hsu C.L., Lo Y.C., Wang Hsu G.S., Kao C.F. (2013). Resveratrol activates the histone H2B ubiquitin ligase, RNF20, in MDA-MB-231 breast cancer cells. J. Funct. Foods.

[131] Luo H., Yang A., Schulte B.A., Wargovich M.J., Wang G.Y. (2013). Resveratrol induces premature senescence in lung cancer cells via ROS-mediated DNA damage. PLoS ONE.

[132] Gao Z., Xu M.S., Barnett T.L., Xu C.W. (2011). Resveratrol induces cellular senescence with attenuated mono-ubiquitination of histone H2B in glioma cells. Biochem. Biophys. Res. Commun..

[133] Chaffer C.L., Weinberg R. (2011). A perspective on cancer cell metastasis. Science.

[134] Eccles S.A., Welch D.R. (2007). Metastasis: Recent discoveries and novel treatment strategies. Lancet.

[135] Wan L., Pantel K., Kang Y. (2013). Tumor metastasis: Moving new biological insights into the clinic. Nat. Med..

[136] Steeg P., Theodorescu D. (2008). Metastasis: A therapeutic target for cancer. Nat. Clin. Pract. Oncol..

[137] Steeg P.S. (2016). Targeting metastasis. Nat. Rev. Cancer.

[138] Sleeman J., Steeg P.S. (2010). Metastasis: A therapeutic target for cancer. Eur. J. Cancer.

[139] Fidler I.J. (2003). The pathogenesis of cancer metastasis: The “seed and soil” hypothesis revisited. Nat. Rev. Cancer.

[140] Yang W., Zou L., Huang C., Lei Y. (2014). Redox regulation of cancer metastasis: Molecular signaling and therapeutic opportunities. Drug Dev. Res..

[141] Sun Y., Ma L. (2015). The emerging molecular machinery and therapeutic targets of metastasis. Trends Pharmacol. Sci..

[142] Weiss L. (2000). The molecular genetics of progression and metastasis. Cancer Metastasis Rev..

[143] Shehzad A., Wahid F., Lee Y.S. (2010). Curcumin in cancer chemoprevention: Molecular targets, pharmacokinetics, bioavailability, and clinical trials. Arch. Pharm..

[144] Pulido-Moran M., Moreno-Fernandez J., Ramirez-Tortosa C., Ramirez-Tortosa M.C. (2016). Curcumin and health. Molecules.

[145] Shanmugam M.K., Rane G., Kanchi M.M., Arfuso F., Chinnathambi A., Zayed M.E., Alharbi S.A., Tan B.K.H., Kumar A.P., Sethi G. (2015). The Multifaceted Role of Curcumin in Cancer Prevention and Treatment. Molecules.

[146] Shehzad A., Lee J., Lee Y.S. (2013). Curcumin in various cancers. BioFactors.

[147] Gupta S.C., Prasad S., Kim J.H., Patchva S., Webb L.J., Priyadarsini I.K., Aggarwal B.B. (2011). Multitargeting by curcumin as revealed by molecular interaction studies. Nat. Prod. Rep..

[148] Aggarwal S., Ichikawa H., Takada Y., Sandur S.K., Shishodia S., Aggarwal B.B. (2006). Curcumin (Diferuloylmethane) down-regulates expression of cell proliferation and antiapoptotic and metastatic gene products through suppression of IκBα Kinase and Akt activation. Mol. Pharmacol..

[149] Bachmeier B.E., Mohrenz I.V., Mirisola V., Schleicher E., Romeo F., Höhneke C., Jochum M., Nerlich A.G., Pfeffer U. (2008). Curcumin downregulates the inflammatory cytokines CXCL1 and -2 in breast cancer cells via NFκB. Carcinogenesis.

[150] Killian P.H., Kronski E., Michalik K.M., Barbieri O., Astigiano S., Sommerhoff C.P., Pfeffer U., Nerlich A.G., Bachmeier B.E. (2012). Curcumin inhibits prostate cancer metastasis in vivo by targeting the inflammatory cytokines CXCL1 and -2. Carcinogenesis.

[151] Minn A.J., Gupta G.P., Siegel P.M., Bos P.D., Shu W., Giri D.D., Viale A., Olshen A.B., Gerald W.L., Massague J. (2005). Genes that mediate breast cancer metastasis to lung. Nature.

[152] Narasimhan M., Ammanamanchi S. (2008). Curcumin blocks RON tyrosine kinase-mediated invasion of breast carcinoma cells. Cancer Res..

[153] Thangasamy A., Rogge J., Ammanamanchi S. (2009). Recepteur d’Origine nantais tyrosine kinase is a direct target of hypoxia-inducible factor-1α-mediated invasion of breast carcinoma cells. J. Biol. Chem..

[154] Zong H., Wang F., Fan Q.-X., Wang L.-X. (2012). Curcumin inhibits metastatic progression of breast cancer cell through suppression of urokinase-type plasminogen activator by NF-kappa B signaling pathways. Mol. Biol. Rep..

[155] Woo M.S., Jung S.H., Kim S.Y., Hyun J.W., Ko K.H., Kim W.K., Kim H.S. (2005). Curcumin suppresses phorbol ester-induced matrix metalloproteinase-9 expression by inhibiting the PKC to MAPK signaling pathways in human astroglioma cells. Biochem. Biophys. Res. Commun..

[156] Shankar S., Ganapathy S., Chen Q., Srivastava R.K. (2008). Curcumin sensitizes TRAIL-resistant xenografts: Molecular mechanisms of apoptosis, metastasis and angiogenesis. Mol. Cancer.

[157] Chen M.-C., Chang W.-W., Kuan Y.-D., Lin S.-T., Hsu H.-C., Lee C.-H. (2012). Resveratrol inhibits LPS-induced epithelial-mesenchymal transition in mouse melanoma model. Innate Immun..

[158] Kim Y.S., Sull J.W., Sung H.J. (2012). Suppressing effect of resveratrol on the migration and invasion of human metastatic lung and cervical cancer cells. Mol. Biol. Rep..

[159] Liu P.L., Tsai J.R., Charles A.L., Hwang J.J., Chou S.H., Ping Y.H., Lin F.Y., Chen Y.L., Hung C.Y., Chen W.C. (2010). Resveratrol inhibits human lung adenocarcinoma cell metastasis by suppressing heme oxygenase 1-mediated nuclear factor-κB pathway and subsequently downregulating expression of matrix metalloproteinases. Mol. Nutr. Food Res..

[160] Dulak J., Łoboda A., Zagórska A., Józkowicz A. (2004). Complex role of heme oxygenase-1 in angiogenesis. Antioxid. Redox Signal..

[161] Yu H., Pan C., Zhao S., Wang Z., Zhang H., Wu W. (2008). Resveratrol inhibits tumor necrosis factor-alpha-mediated matrix metalloproteinase-9 expression and invasion of human hepatocellular carcinoma cells. Biomed. Pharmacother..

[162] Ryu J., Ku B.M., Lee Y.K., Jeong J.Y., Kang S., Choi J., Yang Y., Lee D.H., Roh G.S., Kim H.J. (2011). Resveratrol reduces TNF-alpha-induced U373MG human glioma cell invasion through regulating NF-kappaB activation and uPA/uPAR expression. Anticancer Res..

[163] Behrens J. (1993). The role of cell adhesion molecules in cancer invasion and metastasis. Breast Cancer Res. Treat..

[164] Reymond N., D’Água B.B., Ridley A.J. (2013). Crossing the endothelial barrier during metastasis. Nat. Rev. Cancer.

[165] Okegawa T., Pong R.-C., Li Y., Hsieh J.-T. (2004). The role of cell adhesion molecule in cancer progression and its application in cancer therapy. Acta Biochim. Pol..

[166] Park J.S., Kim K.M., Kim M.H., Chang H.J., Baek M.K., Kim S.M., Jung Y. (2009). Do resveratrol inhibits tumor cell adhesion to endothelial cells by blocking ICAM-1 expression. Anticancer Res..

[167] Visse R., Nagase H. (2003). Matrix metalloproteinases and tissue inhibitors of metalloproteinases: Structure, function, and biochemistry. Circ. Res..

[168] Kumar D., Kumar M., Saravanan C., Singh S.K. (2012). Curcumin: A potential candidate for matrix metalloproteinase inhibitors. Expert Opin. Ther. Targets.

[169] Brown G.T., Murray G.I. (2015). Current mechanistic insights into the roles of matrix metalloproteinases in tumour invasion and metastasis. J. Pathol..

[170] Choe G., Park J.K., Jouben-Steele L., Kremen T.J., Liau L.M., Vinters H.V., Cloughesy T.F., Mischel P.S. (2002). Active matrix metalloproteinase 9 expression is associated with primary glioblastoma subtype. Clin. Cancer Res..

[171] Roomi M.W., Monterrey J.C., Kalinovsky T., Rath M., Niedzwiecki A. (2009). Patterns of MMP-2 and MMP-9 expression in human cancer cell lines. Oncol. Rep..

[172] Jezierska A., Motyl T. (2009). Matrix metalloproteinase-2 involvement in breast cancer progression: A mini-review. Med. Sci. Monit..

[173] Xu X., Wang Y., Chen Z., Sternlicht M.D., Hidalgo M., Steffensen B. (2005). Matrix metalloproteinase-2 contributes to cancer cell migration on collagen. Cancer Res..

[174] Chen Q., Gao Q., Chen K., Wang Y., Chen L., Li X. (2015). Curcumin suppresses migration and invasion of human endometrial carcinoma cells. Oncol. Lett..

[175] Lakka S.S., Jasti S.L., Gondi C., Boyd D., Chandrasekar N., Dinh D.H., Olivero W.C., Gujrati M., Rao J.S. (2002). Downregulation of MMP-9 in ERK-mutated stable transfectants inhibits glioma invasion in vitro. Oncogene.

[176] Cheng T.S., Chen W.C., Lin Y.Y., Tsai C.H., Liao C.I., Shyu H.Y., Ko C.J., Tzeng S.F., Huang C.Y., Yang P.C. (2013). Curcumin-targeting pericellular serine protease matriptase role in suppression of prostate cancer cell invasion, tumor growth, and metastasis. Cancer Prev. Res..

[177] Fan Z., Duan X., Cai H., Wang L., Li M., Qu J., Li W., Wang Y., Wang J. (2015). Curcumin inhibits the invasion of lung cancer cells by modulating the PKCα/Nox-2/ROS/ATF-2/MMP-9 signaling pathway. Oncol. Rep..

[178] Chen Q.-Y., Zheng Y., Jiao D.-M., Chen F.-Y., Hu H.-Z., Wu Y.-Q., Song J., Yan J., Wu L.-J., Lv G.-Y. (2014). Curcumin inhibits lung cancer cell migration and invasion through Rac1-dependent signaling pathway. J. Nutr. Biochem..

[179] Banerji A., Chakrabarti J., Mitra A., Chatterjee A. (2004). Effect of curcumin on gelatinase a (MMP-2) activity in B16F10 melanoma cells. Cancer Lett..

[180] Kurschat P., Zigrino P., Nischt R., Breitkopf K., Steurer P., Klein C.E., Krieg T., Mauch C. (1999). Tissue inhibitor of matrix metalloproteinase-2 regulates matrix metalloproteinase-2 activation by modulation of membrane-type 1 matrix metalloproteinase activity in high and low invasive melanoma cell lines. J. Biol. Chem..

[181] Sieg D.J., Hauck C.R., Ilic D., Klingbeil C.K., Schaefer E., Damsky C.H., Schlaepfer D.D. (2000). FAK integrates growth-factor and integrin signals to promote cell migration. Nat. Cell Biol..

[182] Mitra A., Chakrabarti J., Banerji A., Chatterjee A., Das B.R. (2006). Curcumin, a potential inhibitor of MMP-2 in human laryngeal squamous carcinoma cells HEp2. J. Environ. Pathol. Toxicol. Oncol..

[183] Liao H., Wang Z., Deng Z., Ren H., Li X. (2015). Curcumin inhibits lung cancer invasion and metastasis by attenuating GLUT1/MT1-MMP/MMP2 pathway. Int. J. Clin. Exp. Med..

[184] Gagliano N., Moscheni C., Torri C., Magnani I., Bertelli A.A., Gioia M. (2005). Effect of resveratrol on matrix metalloproteinase-2 (MMP-2) and Secreted Protein Acidic and Rich in Cysteine (SPARC) on human cultured glioblastoma cells. Biomed. Pharmacother..

[185] Gunther S., Ruhe C., Derikito M.G., Bose G., Sauer H., Wartenberg M. (2007). Polyphenols prevent cell shedding from mouse mammary cancer spheroids and inhibit cancer cell invasion in confrontation cultures derived from embryonic stem cells. Cancer Lett..

[186] Lee H.S., Ha A.W., Kim W.K. (2012). Effect of resveratrol on the metastasis of 4T1 mouse breast cancer cells in vitro and in vivo. Nutr. Res. Pract..

[187] Sun C., Hu Y., Guo T., Wang H., Zhang X., He W., Tan H. (2006). Resveratrol as a novel agent for treatment of multiple myeloma with matrix metalloproteinase inhibitory activity. Acta Pharmacol. Sin..

[188] Weng C.J., Wu C.F., Huang H.W., Wu C.H., Ho C.T., Yen G.C. (2010). Evaluation of anti-invasion effect of resveratrol and related methoxy analogues on human hepatocarcinoma cells. J. Agric. Food Chem..

[189] Cavallaro U., Christofori G. (2004). Cell adhesion and signalling by cadherins and Ig-CAMs in cancer. Nat. Rev. Cancer.

[190] Larue L., Antos C., Butz S., Huber O., Delmas V., Dominis M., Kemler R. (1996). A role for cadherins in tissue formation. Development.

[191] Zhang B., Zhang H., Shen G. (2015). Metastasis-associated protein 2 (MTA2) promotes the metastasis of non-small-cell lung cancer through the inhibition of the cell adhesion molecule Ep-CAM and E-cadherin. Jpn. J. Clin. Oncol..

[192] Galván J.A., Zlobec I., Wartenberg M., Lugli A., Gloor B., Perren A., Karamitopoulou E. (2015). Expression of E-cadherin repressors SNAIL, ZEB1 and ZEB2 by tumour and stromal cells influences tumour-budding phenotype and suggests heterogeneity of stromal cells in pancreatic cancer. Br. J. Cancer.

[193] Luo S.-L., Xie Y.-G., Li Z., Ma J.-H., Xu X. (2014). E-cadherin expression and prognosis of oral cancer: A meta-analysis. Tumour Biol..

[194] Schneider M.R., Hiltwein F., Grill J., Blum H., Krebs S., Klanner A., Bauersachs S., Bruns C., Longerich T., Horst D. (2014). Evidence for a role of E-cadherin in suppressing liver carcinogenesis in mice and men. Carcinogenesis.

[195] Liu X., Chu K.-M., Liu X., Chu K.-M. (2014). E-cadherin and gastric cancer: Cause, consequence, and applications. Biomed. Res. Int..

[196] Barber A.G., Castillo-Martin M., Bonal D.M., Jia A.J., Rybicki B.A., Christiano A.M., Cordon-Cardo C. (2015). PI3K/AKT pathway regulates E-cadherin and Desmoglein 2 in aggressive prostate cancer. Cancer Med..

[197] Trillsch F., Kuerti S., Eulenburg C., Burandt E., Woelber L., Prieske K., Eylmann K., Oliveira-Ferrer L., Milde-Langosch K., Mahner S. (2016). E-Cadherin fragments as potential mediators for peritoneal metastasis in advanced epithelial ovarian cancer. Br. J. Cancer.

[198] Vergara D., Simeone P., Latorre D., Cascione F., Leporatti S., Trerotola M., Giudetti A.M., Capobianco L., Lunetti P., Rizzello A. (2015). Proteomics analysis of E-cadherin knockdown in epithelial breast cancer cells. J. Biotechnol..

[199] Xu W., Kimelman D. (2007). Mechanistic insights from structural studies of beta-catenin and its binding partners. J. Cell Sci..

[200] Chen H.-W., Lee J.-Y., Huang J.-Y., Wang C.-C., Chen W.-J., Su S.-F., Huang C.-W., Ho C.-C., Chen J.J.W., Tsai M.-F. (2008). Curcumin inhibits lung cancer cell invasion and metastasis through the tumor suppressor HLJ1. Cancer Res..

[201] Calderwood S.K., Khaleque M.A., Sawyer D.B., Ciocca D.R. (2006). Heat shock proteins in cancer: Chaperones of tumorigenesis. Trends Biochem. Sci..

[202] Chen C.C., Sureshbabul M., Chen H.W., Lin Y.S., Lee J.Y., Hong Q.S., Yang Y.C., Yu S.L. (2013). Curcumin suppresses metastasis via Sp-1, FAK inhibition, and E-cadherin upregulation in colorectal cancer. Evid. Based Complement. Altern. Med..

[203] Kalluri R., Weinberg R.A. (2009). The basics of epithelial-mesenchymal transition. J. Clin. Investig..

[204] Tan C., Zhang L., Cheng X., Lin X.-F.F., Lu R.-R.R., Bao J.-D.D., Yu H.-X.X. (2015). Curcumin inhibits hypoxia-induced migration in K1 papillary thyroid cancer cells. Exp. Biol. Med..

[205] Zhang C.-Y., Zhang L., Yu H.-X., Bao J.-D., Lu R.-R. (2013). Curcumin inhibits the metastasis of K1 papillary thyroid cancer cells via modulating E-cadherin and matrix metalloproteinase-9 expression. Biotechnol. Lett..

[206] Zhang L., Cheng X., Gao Y., Zhang C., Bao J., Guan H., Yu H., Lu R., Xu Q., Sun Y. (2016). Curcumin inhibits metastasis in human papillary thyroid carcinoma BCPAP cells via down-regulation of the TGF-β/Smad2/3 signaling pathway. Exp. Cell Res..

[207] Zhang Z., Chen H., Xu C., Song L., Huang L., Lai Y., Wang Y., Chen H., Gu D., Ren L., Yao Q. (2016). Curcumin inhibits tumor epithelial-mesenchymal transition by downregulating the Wnt signaling pathway and upregulating NKD2 expression in colon cancer cells. Oncol. Rep..

[208] Du Y., Long Q., Zhang L., Shi Y., Liu X., Li X., Guan B., Tian Y., Wang X., Li L., He D. (2015). Curcumin inhibits cancer-associated fibroblast-driven prostate cancer invasion through MAOA/mTOR/HIF-1α signaling. Int. J. Oncol..

[209] Zhao G., Han X., Zheng S., Li Z., Sha Y., Ni J., Sun Z., Qiao S., Song Z. (2016). Curcumin induces autophagy, inhibits proliferation and invasion by downregulating AKT/mTOR signaling pathway in human melanoma cells. Oncol. Rep..

[210] Guan F., Ding Y., Zhang Y., Zhou Y., Li M., Wang C. (2016). Curcumin suppresses proliferation and migration of MDA-MB-231 breast cancer cells through autophagy-dependent Akt degradation. PLoS ONE.

[211] Guo H., Tian T., Nan K., Wang W. (2010). p57: A multifunctional protein in cancer (Review). Int. J. Oncol..

[212] Dhillon A.S., Hagan S., Rath O., Kolch W. (2007). MAP kinase signalling pathways in cancer. Oncogene.

[213] Park S.Y., Jeong K.J., Lee J., Yoon D.S., Choi W.S., Kim Y.K., Han J.W., Kim Y.M., Kim B.K., Lee H.Y. (2007). Hypoxia enhances LPA-induced HIF-1α and VEGF expression: Their inhibition by resveratrol. Cancer Lett..

[214] Baribeau S., Chaudhry P., Parent S., Asselin E. (2014). Resveratrol inhibits cisplatin-induced epithelial-to- mesenchymal transition in ovarian cancer cell lines. PLoS ONE.

[215] Lin F., Hsieh Y., Yang S., Chen C., Tang C., Chuang Y., Lin C., Chen M. (2015). Resveratrol suppresses TPA-induced matrix metalloproteinase-9 expression through the inhibition of MAPK pathways in oral cancer cells. J. Oral Pathol. Med..

[216] Sun T., Chen Q.Y., Wu L.J., Yao X.M., Sun X.J. (2012). Antitumor and antimetastatic activities of grape skin polyphenols in a murine model of breast cancer. Food Chem. Toxicol..

[217] Tang F., Chiang E.I., Sun Y. (2008). Resveratrol inhibits heregulin-β 1-mediated matrix metalloproteinase-9 expression and cell invasion in human breast cancer cells. J. Nutr. Biochem..

[218] Gweon E.J., Kim S.J. (2013). Resveratrol induces MMP-9 and cell migration via the p38 kinase and PI-3K pathways in HT1080 human fbrosarcoma cells. Oncol. Rep..

[219] Yeh C.B., Hsieh M.J., Lin C.W., Chiou H.L., Lin P.Y., Chen T.Y., Yang S.F. (2013). The antimetastatic effects of resveratrol on hepatocellular carcinoma through the downregulation of a metastasis-associated protease by SP-1 modulation. PLoS ONE.

[220] Yang S., Lee W., Tan P., Tang C., Hsiao M., Hsieh F., Chien M. (2015). Upregulation of miR-328 and inhibition of CREB-DNA-binding activity are critical for resveratrol-mediated suppression of matrix metalloproteinase-2 and subsequent metastatic ability in human osteosarcomas. Oncotarget.

[221] Vara J.Á.F., Casado E., de Castro J., Cejas P., Belda-Iniesta C., González-Barón M. (2004). PI3K/Akt signalling pathway and cancer. Cancer Treat. Rev..

[222] Altomare D.A., Testa J.R. (2005). Perturbations of the AKT signaling pathway in human cancer. Oncogene.

[223] Bhattacharya S., Darjatmoko S.R., Polans A.S. (2011). Resveratrol modulates the malignant properties of cutaneous melanoma through changes in the activation and attenuation of the antiapoptotic protooncogenic protein Akt/PKB. Melanoma Res..

[224] Jiao Y., Li H., Liu Y., Guo A., Xu X., Qu X., Wang S., Zhao J., Li Y., Cao Y. (2015). Resveratrol inhibits the invasion of glioblastoma-initiating cells via down-regulation of the PI3K/Akt/NF-κB signaling pathway. Nutrients.

[225] Li W., Ma J., Ma Q., Li B., Han L., Liu J., Xu Q., Duan W., Yu S., Wang F., Wu E. (2013). Resveratrol inhibits the epithelial-mesenchymal transition of pancreatic cancer cells via suppression of the PI-3K/Akt/NF-κB pathway. Curr. Med. Chem..

[226] Tang F.Y., Su Y.C., Chen N.C., Hsieh H.S., Chen K.S. (2008). Resveratrol inhibits migration and invasion of human breast-cancer cells. Mol. Nutr. Food Res..

[227] Kalinski T., Sel S., Hutten H., Ropke M., Roessner A., Nass N. (2014). Curcumin blocks interleukin-1 signaling in chondrosarcoma cells. PLoS ONE.

[228] Da W., Zhu J., Wang L., Sun Q. (2015). Curcumin suppresses lymphatic vessel density in an in vivo human gastric cancer model. Tumour Biol..

[229] Rubin L.L., de Sauvage F.J. (2006). Targeting the Hedgehog pathway in cancer. Nat. Rev. Drug Discov..

[230] Amakye D., Jagani Z., Dorsch M. (2013). Unraveling the therapeutic potential of the Hedgehog pathway in cancer. Nat. Med..

[231] Gupta S., Takebe N., LoRusso P. (2010). Targeting the Hedgehog pathway in cancer. Ther. Adv. Med. Oncol..

[232] Li J., Chong T., Wang Z., Chen H., Li H., Cao J., Zhang P., Li H. (2014). A novel anti-cancer effect of resveratrol: Reversal of epithelial-mesenchymal transition in prostate cancer cells. Mol. Med. Rep..

[233] Matise M.P., Joyner A.L. (1999). Gli genes in development and cancer. Oncogene.

[234] Gao Q., Yuan Y., Gan H., Peng Q. (2015). Resveratrol inhibits the hedgehog signaling pathway and epithelial-mesenchymal transition and suppresses gastric cancer invasion and metastasis. Oncol. Lett..

[235] Li W., Cao L., Chen X., Lei J., Ma Q. (2016). Resveratrol inhibits hypoxia-driven ROS-induced invasive and migratory ability of pancreatic cancer cells via suppression of the Hedgehog signaling pathway. Oncol. Rep..

[236] Yu H., Lee H., Herrmann A., Buettner R., Jove R. (2014). Revisiting STAT3 signalling in cancer: New and unexpected biological functions. Nat. Rev. Cancer.

[237] Siveen K.S., Sikka S., Surana R., Daia X., Zhang J., Kumar A.P., Tan B.K.H., Sethi G., Bishayee A. (2014). Targeting the STAT-3 signaling pathway in cancer: Role of synthetic and natural inhibitors. Biochim. Biophys. Acta.

[238] Eswaran D., Huang S. (2009). STAT-3 as a central regulator of tumor metastases. Curr. Mol. Med..

[239] Lee-Chang C., Bodogai M., Martin-Montalvo A., Wejksza K., Sanghvi M., Moaddel R., de Cabo R., Biragyn A. (2013). Inhibition of breast cancer metastasis by resveratrol-mediated inactivation of tumor-evoked regulatory B cells. J. Immunol..

[240] Olkhanud P.B., Damdinsuren B., Bodogai M., Gress R.E., Sen R., Wejksza K., Malchinkhuu E., Wersto R.P., Biragyn A. (2011). Tumor-evoked regulatory B cells promote breast cancer metastasis by converting resting CD4^+^ T cells to T-regulatory cells. Cancer Res..

[241] Kimura Y., Sumiyoshi M. (2016). Resveratrol prevents tumor growth and metastasis by inhibiting lymphangiogenesis and M2 macrophage activation and differentiation in tumor-associated macrophages. Nutr. Cancer.

[242] Schmieder A., Michel J., Schönhaar K., Goerdt S., Schledzewski K. (2012). Differentiation and gene expression profile of tumor-associated macrophages. Semin. Cancer Biol..

[243] Wang H., Feng H., Zhang Y. (2016). Resveratrol inhibits hypoxia-induced glioma cell migration and invasion by the p-STAT-3/miR-34a axis. Neoplasma.

[244] Misso G., Di Martino M.T., De Rosa G., Farooqi A.A., Lombardi A., Campani V., Zarone M.R., Gullà A., Tagliaferri P., Tassone P. (2014). Mir-34: A New Weapon Against Cancer?. Mol. Ther. Nucleic Acids.

[245] Chen Q.Y., Jiao D.E.M., Yao Q.H., Yan J., Song J., Chen F.Y., Lu G.H., Zhou J.Y. (2012). Expression analysis of Cdc42 in lung cancer and modulation of its expression by curcumin in lung cancer cell lines. Int. J. Oncol..

[246] Sahai E., Marshall C.J. (2002). RHO-GTPases and cancer. Nat. Rev. Cancer.

[247] Wang L., Dong Z., Zhang Y., Miao J. (2012). The roles of integrin β4 in vascular endothelial cells. J. Cell. Physiol..

[248] Coleman D.T., Soung Y.H., Surh Y.J., Cardelli J.A., Chung J. (2015). Curcumin prevents palmitoylation of integrin β4 in breast cancer cells. PLoS ONE.

[249] Dorai T., Diouri J., O’Shea O., Doty S.B. (2014). Curcumin inhibits prostate cancer bone metastasis by up-regulating bone morphogenic protein-7 in vivo. J. Cancer Ther..

[250] Buijs J.T., Rentsch C.A., van der Horst G., van Overveld P.G.M., Wetterwald A., Schwaninger R., Henriquez N.V., Ten Dijke P., Borovecki F., Markwalder R. (2007). BMP7, a putative regulator of epithelial homeostasis in the human prostate, is a potent inhibitor of prostate cancer bone metastasis in vivo. Am. J. Pathol..

[251] Mudduluru G., George-William J.N., Muppala S., Asangani I.A., Kumarswamy R., Nelson L.D., Allgayer H. (2011). Curcumin regulates miR-21 expression and inhibits invasion and metastasis in colorectal cancer. Biosci. Rep..

[252] Selcuklu S.D., Donoghue M.T., Spillane C. (2009). miR-21 as a key regulator of oncogenic processes. Biochem. Soc. Trans..

[253] Yeh W., Lin H., Huang C., Huang B. (2015). Migration-prone glioma cells show curcumin resistance associated with enhanced expression of miR-21 and invasion/anti-apoptosis-related proteins. Oncotarget.

[254] Bessette D.C., Wong P.C.W., Pallen C.J. (2007). PRL-3: A metastasis-associated phosphatase in search of a function. Cells Tissues Organs.

[255] Rouleau C., Roy A., St. Martin T., Dufault M.R., Boutin P., Liu D., Zhang M., Puorro-Radzwill K., Rulli L., Reczek D. (2006). Protein tyrosine phosphatase PRL-3 in malignant cells and endothelial cells: Expression and function. Mol. Cancer Ther..

[256] Wang L., Shen Y., Song R., Sun Y., Xu J., Xu Q. (2009). An anticancer effect of curcumin mediated by down-regulating phosphatase of regenerating liver-3 expression on highly metastatic melanoma cells. Mol. Pharmacol..

[257] Dhar S., Kumar A., Li K., Tzivion G., Levenson A.S. (2015). Resveratrol regulates PTEN/Akt pathway through inhibition of MTA1/HDAC unit of the NuRD complex in prostate cancer. Biochim. Biophys. Acta Mol. Cell Res..

[258] Jeong K.J., Cho K.H., Panupinthu N., Kim H., Kang J., Park C.G., Mills G.B., Lee H.Y. (2013). EGFR mediates LPA-induced proteolytic enzyme expression and ovarian cancer invasion: Inhibition by resveratrol. Mol. Oncol..

[259] Ji Q., Liu X., Fu X., Zhang L., Sui H., Zhou L., Sun J., Cai J., Qin J., Ren J., Li Q. (2013). Resveratrol inhibits invasion and metastasis of colorectal cancer cells via MALAT1 mediated Wnt/β-catenin signal pathway. PLoS ONE.

[260] Ji Q., Liu X., Han Z., Zhou L., Sui H., Yan L., Jiang H., Ren J., Cai J., Li Q. (2015). Resveratrol suppresses epithelial-to-mesenchymal transition in colorectal cancer through TGF-β1/Smads signaling pathway mediated Snail/E-cadherin expression. BMC Cancer.

[261] Mikula-Pietrasik J., Sosinska P., Ksiazek K. (2014). Resveratrol inhibits ovarian cancer cell adhesion to peritoneal mesothelium in vitro by modulating the production of α5β1 integrins and hyaluronic acid. Gynecol. Oncol..

[262] Rodrigue C.M., Porteu F., Navarro N., Bruyneel E., Bracke M., Romeo P.-H., Gespach C., Garel M.-C. (2005). The cancer chemopreventive agent resveratrol induces tensin, a cell–matrix adhesion protein with signaling and antitumor activities. Oncogene.

[263] Wang H., Zhang H., Tang L., Chen H., Wu C., Zhao M., Yang Y., Chen X., Liu G. (2013). Resveratrol inhibits TGF-β1-induced epithelial-to-mesenchymal transition and suppresses lung cancer invasion and metastasis. Toxicology.

[264] Zykova T.A., Zhu F., Zhai X., Ma W.Y., Ermakova S.P., Ki W.L., Bode A.M., Dong Z. (2008). Resveratrol directly targets COX-2 to inhibit carcinogenesis. Mol. Carcinog..

[265] Salado C., Olaso E., Gallot N., Valcarcel M., Egilegor E., Mendoza L., Vidal-Vanaclocha F. (2011). Resveratrol prevents inflammation-dependent hepatic melanoma metastasis by inhibiting the secretion and effects of interleukin-18. J. Transl. Med..

[266] Green D.R., Kroemer G. (2004). The pathophysiology of mitochondrial cell death. Science.

[267] Henry-Mowatt J., Dive C., Martinou J., James D. (2004). Role of mitochondrial membrane permeabilization in apoptosis and cancer. Oncogene.

[268] Zou H., Henzel W.J., Liu X., Lutschg A., Wang X. (1997). Apaf-1, a human protein homologous to *C. elegans* CED-4, participates in cytochrome c dependent activation of caspace 3. Cell.

[269] Adrain C., Slee E.A., Harte M.T., Martin S.J. (1999). Regulation of apoptotic protease activating factor-1 oligomerization and apoptosis by the WD-40 repeat region. J. Biol. Chem..

[270] Kroemer G., Galluzzi L., Brenner C. (2007). Mitochondrial membrane permeabilization in cell death. Physiol. Rev..

[271] Bratton S.B., Walker G., Srinivasula S.M., Sun X.M., Butterworth M., Alnemri E.S., Cohen G.M. (2001). Recruitment, activation and retention of caspases-9 and-3 by Apaf-1 apoptosome and associated XIAP complexes. EMBO J..

[272] Fulda S., Debatin K.-M. (2006). Extrinsic versus intrinsic apoptosis pathways in anticancer chemotherapy. Oncogene.

[273] Susin S.A., Zamzami N., Castedo M., Hirsch T., Marchetti P., Macho A., Daugas E., Geuskens M., Kroemer G. (1996). Bcl-2 inhibits the mitochondrial release of an apoptogenic protease. J. Exp. Med..

[274] Lin X., Wu G., Huo W.Q., Zhang Y., Jin F.S. (2012). Resveratrol induces apoptosis associated with mitochondrial dysfunction in bladder carcinoma cells. Int. J. Urol..

[275] Van Ginkel P.R., Sareen D., Subramanian L., Walker Q., Darjatmoko S.R., Lindstrom M.J., Kulkarni A., Albert D.M., Polans A.S. (2007). Resveratrol inhibits tumor growth of human neuroblastoma and mediates apoptosis by directly targeting mitochondria. Clin. Cancer Res..

[276] Alkhalaf M., El-Mowafy A., Renno W., Rachid O., Ali A., Al-Attyiah R. (2008). Resveratrol-induced apoptosis in human breast cancer cells is mediated primarily through the caspase-3-dependent pathway. Arch. Med. Res..

[277] Zhang W., Fei Z., Zhen H.-N., Zhang J.-N., Zhang X. (2007). Resveratrol inhibits cell growth and induces apoptosis of rat C6 glioma cells. J. Neurooncol..

[278] Zhang W., Wang X., Chen T. (2011). Resveratrol induces mitochondria-mediated AIF and to a lesser extent caspase-9-dependent apoptosis in human lung adenocarcinoma ASTC-a-1 cells. Mol. Cell. Biochem..

[279] Zhang W., Wang X., Chen T. (2012). Resveratrol induces apoptosis via a Bak-mediated intrinsic pathway in human lung adenocarcinoma cells. Cell. Signal..

[280] Qadir M.I., Naqvi S.T.Q., Muhammad S.A. (2016). Curcumin: A polyphenol with molecular targets for cancer control. Asian Pac. J. Cancer Prev..

[281] Song F., Zhang L., Yu H.-X., Lu R.-R., Bao J.-D., Tan C., Sun Z. (2012). The mechanism underlying proliferation-inhibitory and apoptosis-inducing effects of curcumin on papillary thyroid cancer cells. Food Chem..

[282] Khan M.A., Gahlot S., Majumdar S. (2012). Oxidative stress induced by curcumin promotes the death of cutaneous T-cell lymphoma (HuT-78) by disrupting the function of several molecular targets. Mol. Cancer Ther..

[283] Kaushik G., Kaushik T., Yadav S.K., Sharma S.K., Ranawat P., Khanduja K.L., Pathak C.M. (2012). Curcumin sensitizes lung adenocarcinoma cells to apoptosis via intracellular redox status mediated pathway. Indian J. Exp. Biol..

[284] Hussain A.R., Uddin S., Bu R., Khan O.S., Ahmed S.O., Ahmed M., Al-Kuraya K.S. (2011). Resveratrol suppresses constitutive activation of AKT via generation of ROS and induces apoptosis in diffuse large B cell lymphoma cell lines. PLoS ONE.

[285] Wang Z., Li W., Meng X., Jia B. (2012). Resveratrol induces gastric cancer cell apoptosis via reactive oxygen species, but independent of sirtuin1. Clin. Exp. Pharmacol. Physiol..

[286] Su C.C., Lin J.G., Li T.M., Chung J.G., Yang J.S., Ip S.W., Lin W.C., Chen G.W. (2006). Curcumin-induced apoptosis of human colon cancer colo 205 cells through the production of ROS, Ca^2+^ and the activation of caspase-3. Anticancer Res..

[287] Tan T.-W., Tsai H.-R., Lu H.-F., Lin H.-L., Tsou M.-F., Lin Y.-T., Tsai H.-Y., Chen Y.-F., Chung J.-G. (2006). Curcumin-induced cell cycle arrest and apoptosis in human acute promyelocytic leukemia HL-60 cells via MMP changes and caspase-3 activation. Anticancer Res..

[288] Ibrahim A., El-Meligy A., Lungu G., Fetaih H., Dessouki A., Stoica G., Barhoumi R. (2011). Curcumin induces apoptosis in a murine mammary gland adenocarcinoma cell line through the mitochondrial pathway. Eur. J. Pharmacol..

[289] Wang W., Chiang I., Ding K., Chung J.-G., Lin W.-J., Lin S.-S., Hwang J.-J. (2012). Curcumin-induced apoptosis in human hepatocellular carcinoma J5 cells: Critical role of Ca^+2^-dependent pathway. Evid. Based Complement. Altern. Med..

[290] Marchetti C., Ribulla S., Magnelli V., Patrone M., Burlando B. (2016). Resveratrol induces intracellular Ca^2+^ rise via T-type Ca^2+^ channels in a mesothelioma cell line. Life Sci..

[291] Skommer J., Wlodkowic D., Pelkonen J. (2006). Cellular foundation of curcumin-induced apoptosis in follicular lymphoma cell lines. Exp. Hematol..

[292] Guo L., Chen X., Hu Y., Yu Z., Wang D., Liu J. (2013). Curcumin inhibits proliferation and induces apoptosis of human colorectal cancer cells by activating the mitochondria apoptotic pathway. Phyther. Res..

[293] Huang T.Y., Tsai T.H., Hsu C.W., Hsu Y.C. (2010). Curcuminoids suppress the growth and induce apoptosis through caspase-3-dependent pathways in glioblastoma multiforme (GBM) 8401 cells. J. Agric. Food Chem..

[294] Shankar S., Srivastava R.K. (2007). Involvement of Bcl-2 family members, phosphatidylinositol 3′-kinase/AKT and mitochondrial p53 in curcumin (diferulolylmethane)-induced apoptosis in prostate cancer. Int. J. Oncol..

[295] Yu Z., Shah D.M. (2007). Curcumin down-regulates Ets-1 and Bcl-2 expression in human endometrial carcinoma HEC-1-A cells. Gynecol. Oncol..

[296] Yang J., Ning J., Peng L., He D. (2015). Effect of curcumin on Bcl-2 and Bax expression in nude mice prostate cancer. Int. J. Clin. Exp. Pathol..

[297] Huang T., Lin H., Chen C., Lu C., Wei C., Wu T., Liu F., Lai H. (2010). Resveratrol induces apoptosis of human nasopharyngeal carcinoma cells via activation of multiple apoptotic pathways. J. Cell. Physiol..

[298] Wang B., Liu J., Gong Z. (2015). Resveratrol induces apoptosis in K562 cells via the regulation of mitochondrial signaling pathways. Int. J. Clin. Exp. Med..

[299] Cui J., Sun R., Yu Y., Gou S., Zhao G., Wang C. (2010). Antiproliferative effect of resveratrol in pancreatic cancer cells. Phyther. Res..

[300] Fernández-Pérez F., Belchí-Navarro S., Almagro L., Bru R., Pedreño M.A., Gómez-Ros L.V. (2012). Cytotoxic effect of natural trans-resveratrol obtained from elicited vitis vinifera cell cultures on three cancer cell lines. Plant Foods Hum. Nutr..

[301] Ou X., Chen Y., Cheng X., Zhang X., He Q. (2014). Potentiation of resveratrol-induced apoptosis by matrine in human hepatoma HepG2 cells. Oncol. Rep..

[302] Benchimol S. (2001). P53-dependent pathways of apoptosis. Cell Death Differ..

[303] Fridman J.S., Lowe S.W. (2003). Control of apoptosis by p53. Oncogene.

[304] Reuter S., Eifes S., Dicato M., Aggarwal B.B., Diederich M. (2008). Modulation of anti-apoptotic and survival pathways by curcumin as a strategy to induce apoptosis in cancer cells. Biochem. Pharmacol..

[305] Alkhalaf M. (2007). Resveratrol-induced apoptosis is associated with activation of p53 and inhibition of protein translation in T47D human breast cancer cells. Pharmacology.

[306] Ellisen L.W., Bird J., West D.C., Soreng A.L., Reynolds T.C., Smith S.D., Sklar J. (1991). TAN-l, the human homolog of the drosophila notch gene, is broken by chromosomal translocations in T lymphoblastic neoplasms. Cell.

[307] Lin H., Xiong W., Zhang X., Liu B., Zhang W., Zhang Y., Cheng J., Huang H. (2011). Notch-1 activation-dependent p53 restoration contributes to resveratrol-induced apoptosis in glioblastoma cells. Oncol. Rep..

[308] Ozaki T., Nakagawara A. (2005). P73, a sophisticated P53 family member in the cancer world. Cancer Sci..

[309] Moll U.M., Slade N. (2004). P63 and P73: Roles in development and tumor formation. Mol. Cancer Res..

[310] Wang J., Xie H., Gao F., Zhao T., Yang H., Kang B. (2016). Curcumin induces apoptosis in p53-null Hep3B cells through a TAp73/DNp73-dependent pathway. Tumor Biol..

[311] Harper N., Hughes M., MacFarlane M., Cohen G.M. (2003). Fas-associated death domain protein and caspase-8 are not recruited to the tumor necrosis factor receptor 1 signaling complex during tumor necrosis factor-induced apoptosis. J. Biol. Chem..

[312] Wang S., El-Deiry W.S. (2003). TRAIL and apoptosis induction by TNF-family death receptors. Oncogene.

[313] Shankar S., Chen Q., Siddiqui I., Sarva K., Srivastava R.K. (2007). Sensitization of TRAIL-resistant LNCaP cells by resveratrol (3,4′,5 tri-hydroxystilbene): Molecular mechanisms and therapeutic potential. J. Mol. Signal..

[314] Shankar S., Chen Q., Sarva K., Siddiqui I., Srivastava R.K. (2007). Curcumin enhances the apoptosis-inducing potential of TRAIL in prostate cancer cells: Molecular mechanisms of apoptosis, migration and angiogenesis. J. Mol. Signal..

[315] Lee H., Li T., Tsao J., Fong Y., Tang C. (2012). Curcumin induces cell apoptosis in human chondrosarcoma through extrinsic death receptor pathway. Int. Immunopharmacol..

[316] Ko Y., Chang C., Chien H., Wu C., Lin L. (2011). Resveratrol enhances the expression of death receptor Fas/CD95 and induces differentiation and apoptosis in anaplastic large-cell lymphoma cells. Cancer Lett..

[317] Reis-Sobreiro M., Gajate C., Mollinedo F. (2009). Involvement of mitochondria and recruitment of Fas/CD95 signaling in lipid rafts in resveratrol-mediated antimyeloma and antileukemia actions. Oncogene.

[318] Wu S.H., Hang L.W., Yang J.S., Chen H.Y., Lin H.Y., Chiang J.H., Lu C.C., Yang J.L., Lai T.Y., Ko Y.C. (2010). Curcumin induces apoptosis in human non-small cell lung cancer NCI-H460 cells through ER stress and caspase cascade- and mitochondria-dependent pathways. Anticancer Res..

[319] Li L., Aggarwal B.B., Shishodia S., Abbruzzese J., Kurzrock R. (2004). Nuclear factor-kappaB and IkappaB kinase are constitutively active in human pancreatic cells, and their down-regulation by curcumin (diferuloylmethane) is associated with the suppression of proliferation and the induction of apoptosis. Cancer.

[320] Zanotto-Filho A., Braganhol E., Schroder R., De Souza L.H.T., Dalmolin R.J.S., Pasquali M.A.B., Gelain D.P., Battastini A.M.O., Moreira J.C.F. (2011). NFκB inhibitors induce cell death in glioblastomas. Biochem. Pharmacol..

[321] Sun C., Hu Y., Liu X., Wu T., Wang Y., He W., Wei W. (2006). Resveratrol downregulates the constitutional activation of nuclear factor-κΒ in multiple myeloma cells, leading to suppression of proliferation and invasion, arrest of cell cycle, and induction of apoptosis. Cancer Genet. Cytogenet..

[322] Pozo-guisado E., Merino J.M., Mulero-navarro S., Jesús M., Centeno F., Alvarez-barrientos A., Salguero P.M.F. (2005). Resveratrol-induced apoptosis in MCF-7 human breast cancer cells involves a caspase-independent mechanism with downregulation of Bcl-2 and NF-κB. Int. J. Cancer.

[323] Hardwaj A., Sethi G., Vadhan-Raj S., Bueso-Ramos C., Takada Y., Gaur U., Nair A.S., Shishodia S., Aggarwal B.B. (2007). Resveratrol inhibits proliferation, induces apoptosis, and overcomes chemoresistance through down-regulation of STAT-3 and nuclear factor-kappaB-regulated antiapoptotic and cell survival gene products in human multiple myeloma cells. Blood.

[324] Kizhakkayil J., Thayyullathil F., Chathoth S., Hago A., Patel M., Galadari S. (2010). Modulation of curcumin-induced Akt phosphorylation and apoptosis by PI3K inhibitor in MCF-7 cells. Biochem. Biophys. Res. Commun..

[325] Amin A.R.M.R., Haque A., Rahman M.A., Chen Z.G., Khuri F.R., Shin D.M. (2015). Curcumin induces apoptosis of upper aerodigestive tract cancer cells by targeting multiple pathways. PLoS ONE.

[326] Hussain A.R., Al-Rasheed M., Manogaran P.S., Al-Hussein K.A., Platanias L.C., Al Kuraya K., Uddin S. (2006). Curcumin induces apoptosis via inhibition of PI3-kinase/AKT pathway in acute T cell leukemias. Apoptosis.

[327] Zhao Z., Li C., Xi H., Gao Y., Xu D. (2015). Curcumin induces apoptosis in pancreatic cancer cells through the induction of forkhead box O1 and inhibition of the PI3K/Akt pathway. Mol. Med. Rep..

[328] Wong T.F., Takeda T., Li B., Tsuiji K., Kitamura M., Kondo A., Yaegashi N. (2011). Curcumin disrupts uterine leiomyosarcoma cells through AKT-mTOR pathway inhibition. Gynecol. Oncol..

[329] Wong T.F., Takeda T., Li B., Tsuiji K., Kondo A., Tadakawa M., Nagase S., Yaegashi N. (2014). Curcumin targets the AKT-mTOR pathway for uterine leiomyosarcoma tumor growth suppression. Int. J. Clin. Oncol..

[330] Li Y., Liu J., Liu X., Xing K., Wang Y., Li F., Yao L. (2006). Resveratrol-induced cell inhibition of growth and apoptosis in MCF7 human breast cancer cells are associated with modulation of phosphorylated akt and caspase-9. Appl. Biochem. Biotechnol..

[331] Chakraborty P.K., Mustafi S.B., Ganguly S., Chatterjee M., Raha S. (2008). Resveratrol induces apoptosis in K562 (chronic myelogenous leukemia) cells by targeting a key survival protein, heat shock protein 70. Cancer Sci..

[332] Mustafi S.B., Chakraborty P.K., Raha S. (2010). Modulation of AKT and ERK1/2 pathways by resveratrol in chronic myelogenous leukemia (CML) cells results in the downregulation of Hsp70. PLoS ONE.

[333] Sayed D., He M., Hong C., Gao S., Rane S., Yang Z., Abdellatif M. (2010). MicroRNA-21 is a downstream effector of AKT that mediates its antiapoptotic effects via suppression of fas ligand. J. Biol. Chem..

[334] Sheth S., Jajoo S., Kaur T., Mukherjea D., Sheehan K., Rybak L.P., Ramkumar V. (2012). Resveratrol reduces prostate cancer growth and metastasis by inhibiting the Akt/MicroRNA-21 pathway. PLoS ONE.

[335] Dai Z., Lei P., Xie J., Hu Y. (2015). Antitumor effect of resveratrol on chondrosarcoma cells via phosphoinositide 3-kinase/AKT and p38 mitogen-activated protein kinase pathways. Mol. Med. Rep..

[336] Gomez D.E., Armando R.G., Farina H.G., Menna P.L., Cerrudo C.S., Ghiringhelli P.D., Alonso D.F. (2012). Telomere structure and telomerase in health and disease (Review). Int. J. Oncol..

[337] Chakraborty S., Ghosh U., Bhattacharyya N.P., Bhattacharya R.K., Roy M. (2006). Inhibition of telomerase activity and induction of apoptosis by curcumin in K-562 cells. Mutat. Res..

[338] Chakraborty S.M., Ghosh U., Bhattacharyya N.P., Bhattacharya R.K., Dey S., Roy M. (2007). Curcumin-induced apoptosis in human leukemia cell HL-60 is associated with inhibition of telomerase activity. Mol. Cell. Biochem..

[339] Khaw A.K., Hande M.P., Kalthur G., Hande M.P. (2013). Curcumin inhibits telomerase and induces telomere shortening and apoptosis in brain tumour cells. J. Cell. Biochem..

[340] Lanzilli G., Fuggetta M.P., Tricarico M., Cottarelli A., Serafino A., Falchetti R., Ravagnan G., Turriziani M., Adamo R., Franzese O. (2006). Resveratrol down-regulates the growth and telomerase activity of breast cancer cells in vitro. Int. J. Oncol..

[341] Zhai X.-X., Ding J.-C., Tang Z.-M., Li J.-G., Li Y.-C., Yan Y.-H., Sun J.-C., Zhang C.-X. (2016). Effects of resveratrol on the proliferation, apoptosis and telomerase ability of human A431 epidermoid carcinoma cells. Oncol. Lett..

[342] Yu H., Jove R. (2004). The STATs of cancer—New molecular targets come of age. Nat. Rev. Cancer.

[343] Ihle J.N. (1996). STATs: Signal transducers and activators of transcription. Cell.

[344] Blasius R., Reuter S., Henry E., Dicato M., Diederich M. (2006). Curcumin regulates signal transducer and activator of transcription (STAT) expression in K562 cells. Biochem. Pharmacol..

[345] Zhang Y.P., Li Y.Q., Lv Y.T., Wang J.M. (2015). Effect of curcumin on the proliferation, apoptosis, migration, and invasion of human melanoma A375 cells. Genet. Mol. Res..

[346] Zhang C., Li B., Zhang X., Hazarika P., Aggarwal B.B., Duvic M. (2010). Curcumin selectively induces apoptosis in cutaneous T-cell lymphoma cell lines and patients’ PBMCs: Potential role for STAT-3 and NF-kappaB signaling. J. Investig. Dermatol..

[347] Uddin S., Hussain A.R., Manogaran P.S., Al-Hussein K., Platanias L.C., Gutierrez M.I., Bhatia K.G. (2005). Curcumin suppresses growth and induces apoptosis in primary effusion lymphoma. Oncogene.

[348] Mackenzie G.G., Queisser N., Wolfson M.L., Fraga C.G., Adamo A.M., Oteiza P.I. (2008). Curcumin induces cell-arrest and apoptosis in association with the inhibition of constitutively active NF-kappaB and STAT3 pathways in Hodgkin’s lymphoma cells. Int. J. Cancer.

[349] Baek S.H., Ko J., Lee H., Jung J., Kong M., Lee J., Lee J., Chinnathambi A., Zayed M.E., Alharbi S.A. (2016). Resveratrol inhibits STAT-3 signaling pathway through the induction of SOCS-1: Role in apoptosis induction and radiosensitization in head and neck tumor cells. Phytomedicine.

[350] Trung L.Q., Espinoza J.L., Takami A., Nakao S. (2013). Resveratrol induces cell cycle arrest and apoptosis in malignant NK cells via JAK2/STAT-3 pathway inhibition. PLoS ONE.

[351] Wu M.-L. (2014). Short-term resveratrol exposure causes in vitro and in vivo growth inhibition and apoptosis of bladder cancer. PLoS ONE.

[352] Zhong L.-X., Li H., Wu M., Liu X.-Y., Zhong M., Chen X., Liu J., Zhang Y. (2015). Inhibition of STAT3 signaling as critical molecular event in resveratrol-suppressed ovarian cancer cells. J. Ovarian Res..

[353] Ha M., Kim V.N. (2014). Regulation of microRNA biogenesis. Nat. Rev. Mol. Cell Biol..

[354] Cimmino A., Calin G.A., Fabbri M., Iorio M.V., Ferracin M., Shimizu M., Wojcik S.E., Aqeilan R.I., Zupo S., Dono M. (2005). miR-15 and miR-16 induce apoptosis by targeting BCL2. Proc. Natl. Acad. Sci. USA.

[355] Yang J., Cao Y., Sun J., Zhang Y. (2010). Curcumin reduces the expression of Bcl-2 by upregulating miR-15a and miR-16 in MCF-7 cells. Med. Oncol..

[356] Zhang J., Du Y., Wu C., Ren X., Ti X., Shi J., Zhao F., Yin H. (2010). Curcumin promotes apoptosis in human lung adenocarcinoma cells through miR-186 signaling pathway. Oncol. Rep..

[357] Zhang J., Zhang T., Ti X., Shi J., Wu C., Ren X., Yin H. (2010). Curcumin promotes apoptosis in A549/DDP multidrug-resistant human lung adenocarcinoma cells through an miRNA signaling pathway. Biochem. Biophys. Res. Commun..

[358] Li H., Jia Z., Li A. (2013). Resveratrol repressed viability of U251 cells by miR-21 inhibiting of NF-κB pathway. Mol. Cell. Biochem..

[359] Liu P., Liang H., Xia Q., Li P., Kong H., Lei P., Wang S., Tu Z. (2013). Resveratrol induces apoptosis of pancreatic cancers cells by inhibiting miR-21 regulation of BCL-2 expression. Clin. Transl. Oncol..

[360] Zhou C., Ding J.U.N., Wu Y. (2014). Resveratrol induces apoptosis of bladder cancer cells via miR-21 regulation of the Akt/Bcl-2 signaling pathway. Mol. Med. Rep..

[361] Elmore S. (2007). Apoptosis: A review of programmed cell death. Toxicol. Pathol..

[362] Kroemer G., Galluzzi L., Vandenabeele P., Abrams J., Alnemri E., Baehrecke E., Blagosklonny M., El-Deiry W., Golstein P., Green D. (2009). Classification of cell death. Cell Death Differ..

[363] Aoki H., Takada Y., Kondo S., Sawaya R., Aggarwal B.B., Kondo Y. (2007). Evidence that curcumin suppresses the growth of malignant gliomas in vitro and in vivo through induction of autophagy: Role of Akt and extracellular signal-regulated kinase signaling pathways. Mol. Pharmacol..

[364] Mihaylova M.M., Shaw R.J. (2012). The AMPK signalling pathway coordinates cell growth, autophagy and metabolism. Nat. Cell. Biol..

[365] Xiao K., Jiang J., Guan C., Dong C., Wang G., Bai L., Sun J., Hu C., Bai C. (2013). Curcumin induces autophagy via activating the AMPK signaling pathway in lung adenocarcinoma cells. J. Pharmacol. Sci..

[366] Fu Y., Chang H., Peng X., Bai Q., Yi L., Zhou Y., Zhu J., Mi M. (2014). Resveratrol Inhibits Breast Cancer Stem-Like Cells and Induces Autophagy via Suppressing Wnt/b -Catenin Signaling Pathway. PLoS ONE.

[367] Wang M., Yu T., Zhu C., Sun H., Qiu Y., Zhu X., Li J. (2014). Resveratrol triggers protective autophagy through the Ceramide/Akt/mTOR pathway in melanoma B16 cells the ceramide/Akt/mTOR pathway in melanoma B16 cells. Nutr. Cancer.

[368] Zhou C., Zhao X., Li X., Wang C., Zhang X., Liu X., Ding X. (2013). Curcumin inhibits AP-2γ-induced apoptosis in the human malignant testicular germ cells in vitro. Acta Pharmacol. Sin..

[369] Yu T., Ji J., Guo Y. (2013). Biochemical and Biophysical Research Communications MST1 activation by curcumin mediates JNK activation, Foxo3a nuclear translocation and apoptosis in melanoma cells. Biochem. Biophys. Res. Commun..

[370] Wang K., Fan H., Chen Q., Ma G., Zhu M., Zhang X., Zhang Y., Yu J. (2015). Curcumin inhibits aerobic glycolysis and induces mitochondrial-mediated apoptosis through hexokinase II in human colorectal cancer cells in vitro. Anticancer Drugs.

[371] Sun S., Huang H., Huang C., Lin J. (2012). Cycle arrest and apoptosis in MDA-MB-231/Her2 cells induced by curcumin. Eur. J. Pharmacol..

[372] Saha A., Kuzuhara T., Echigo N., Fujii A., Suganuma M., Fujiki H. (2010). Apoptosis of human lung cancer cells by curcumin mediated through up-regulation of “Growth arrest and DNA damage inducible genes 45 and 153”. Biol. Pharm. Bull..

[373] Milacic V., Banerjee S., Landis-piwowar K.R., Sarkar F.H., Majumdar A.P.N., Dou Q.P. (2008). Curcumin inhibits the proteasome activity in human colon cancer cells in vitro and in vivo. Cancer Res..

[374] Liu H., Ke C., Cheng H., Huang C.F., Su C. (2011). Curcumin-induced mitotic spindle defect and cell cycle arrest in human bladder cancer cells occurs partly through inhibition of aurora A. Mol. Pharmacol..

[375] Lee Y., Park S.Y., Kim Y., Park J.O. (2009). Regulatory effect of the AMPK-COX-2 signaling pathway in curcumin-induced apoptosis in HT-29 colon cancer cells. Ann. N. Y. Acad. Sci..

[376] Lee S.J., Langhans S.A. (2012). Anaphase-promoting complex/cyclosome protein Cdc27 is a target for curcumin-induced cell cycle arrest and apoptosis. BMC Cancer.

[377] Lee S.J., Krauthauser C., Maduskuie V., Fawcett P.T., Olson J.M., Rajasekaran S.A. (2011). Curcumin-induced HDAC inhibition and attenuation of medulloblastoma growth in vitro and in vivo. BMC Cancer.

[378] Kao H., Wu C., Won S., Shin J.-W., Liu H.-S., Su C.-L. (2011). Kinase gene expression and subcellular protein expression pattern of protein kinase c isoforms in curcumin-treated human hepatocellular carcinoma hep 3B cells. Plant Foods Hum. Nutr..

[379] Cui J., Meng X., Gao X., Tan G. (2010). Curcumin decreases the expression of Pokemon by suppressing the binding activity of the Sp1 protein in human lung cancer cells. Mol. Biol. Rep..

[380] Banerjee M., Singh P., Panda D. (2010). Curcumin suppresses the dynamic instability of microtubules, activates the mitotic checkpoint and induces apoptosis in MCF-7 cells. FEBS J..

[381] Cao A., Tang Q., Zhou W., Qiu Y. (2014). Ras/ERK signaling pathway is involved in curcumin-induced cell cycle arrest and apoptosis in human gastric carcinoma AGS cells. J. Asian Nat. Prod..

[382] Fan H., Tian W., Ma X. (2014). Curcumin induces apoptosis of HepG2 cells via inhibiting fatty acid synthase. Target. Oncol..

[383] Chatterjee M., Das S., Janarthan M., Ramachandran H.K., Chatterjee M. (2011). Role of 5-lipoxygenase in resveratrol mediated suppression of 7,12-dimethylbenz (α) anthracene-induced mammary carcinogenesis in rats. Eur. J. Pharmacol..

[384] Lin C., Crawford D.R., Lin S., Sebuyira A., Meng R., Westfall E., Tang H., Lin S., Yu P., Davis P.J. (2011). Inducible COX-2-dependent apoptosis in human ovarian cancer cells. Carcinogenesis.

[385] Zhong L.X., Zhan ZX., Hunag Z.H., Feng M., Xiong J.P. (2016). Resveratrol treatment inhibits proliferation of and induces apoptosis in human colon cancer. Med. Sci. Monit..

[386] Chow S., Wang J., Chuang S., Chang Y., Chu W., Chen W., Chen Y. (2010). Resveratrol-induced p53-independent apoptosis of human nasopharyngeal carcinoma cells is correlated with the downregulation of ΔNp63. Cancer Gene Ther..

[387] Dai W., Wang F., Lu J., Xia Y., He L., Chen K., Li J., Li S., Liu T., Zheng Y. (2015). By reducing hexokinase 2, resveratrol induces apoptosis in HCC cells addicted to aerobic glycolysis and inhibits tumor growth in mice. Oncotarget.

[388] Kai L., Samuel S.K., Levenson A.S. (2010). Resveratrol enhances p53 acetylation and apoptosis in prostate cancer by inhibiting MTA1/NuRD complex. Int. J. Cancer.

[389] Lee K.-A., Lee Y.-J., Ban J.O., Lee Y.-J., Lee S., Cho M., Nam H., Hong J.T., Shim J. (2012). The flavonoid resveratrol suppresses growth of human malignant pleural mesothelioma cells through direct inhibition of specificity protein 1. Int. J. Mol. Med..

[390] Li G., He S., Chang L., Lu H., Zhang H., Zhang H., Chiu J. (2011). GADD45α and annexin A1 are involved in the apoptosis of HL-60 induced by resveratrol. Phytomedicine.

[391] Mohapatra P., Ranjan S., Das D., Siddharth S. (2014). Resveratrol mediated cell death in cigarette smoke transformed breast epithelial cells is through induction of p21Waf1 / Cip1 and inhibition of long patch base excision repair pathway. Toxicol. Appl. Pharmacol..

[392] Shi Y., Yang S., Troup S., Lu X., Callaghan S., Park D.S., Xing Y., Yang X. (2011). Resveratrol induces apoptosis in breast cancer cells by E2F1-mediated up-regulation of ASPP1. Oncol. Rep..

[393] Kumar B., Iqbal M.A., Singh R.K., Bamezai R.N.K. (2015). Biochimie resveratrol inhibits TIGAR to promote ROS induced apoptosis and autophagy. Biochimie.

[394] Ahmad K.A., Harris N.H., Johnson A.D., Lindvall H.C.N., Wang G., Ahmed K. (2007). Protein kinase CK2 modulates apoptosis induced by resveratrol and epigallocatechin-3-gallate in prostate cancer cells. Mol. Cancer Ther..

[395] Wang F., Galson D.L., Roodman G.D., Ouyang H. (2011). Resveratrol triggers the pro-apoptotic endoplasmic reticulum stress response and represses pro-survival XBP1 signaling in human multiple myeloma cells. Exp. Hematol..

[396] Seeni A., Takahashi S., Takeshita K., Tang M., Sugiura S., Sato S., Shirai T. (2008). Suppression of prostate cancer growth by resveratrol in the transgenic rat for adenocarcinoma of prostate (TRAP) model. Asian Pac. J. Cancer Prev..

[397] Lee S.C., Chan J., Clement M.V., Pervaiz S. (2006). Functional proteomics of resveratrol-induced colon cancer cell apoptosis: Caspase-6-mediated cleavage of lamin A is a major signaling loop. Proteomics.

[398] Woo K.J., Lee T.J., Lee S.H., Seo J.H., Jeong Y.J., Park J.W., Kwon T.K. (2007). Elevated gadd153/chop expression during resveratrol-induced apoptosis in human colon cancer cells. Biochem. Pharmacol..

[399] Hsu K., Wu C., Huang S., Wu C., Yo Y., Chen Y., Shiau A., Chou C. (2009). Cathepsin L mediates resveratrol-induced autophagy and apoptotic cell death in cervical cancer cells. Autophagy.

[400] Trincheri N.F., Nicotra G., Follo C., Castino R., Isidoro C. (2007). Resveratrol induces cell death in colorectal cancer cells by a novel pathway involving lysosomal cathepsin D. Carcinogenesis.

[401] Whitlock N., Bahn J.H., Lee S.H., Eling T.E., Baek S.J. (2011). Resveratrol-induced apoptosis is mediated by early growth response-1, Krüppel-like factor 4, and activating transcription factor 3. Cancer Prev. Res..

[402] Pandey P.R., Okuda H., Watabe M., Pai S.K. (2011). Resveratrol suppresses growth of cancer stem-like cells by inhibiting fatty acid synthase. Breast Cancer Res. Treat..

[403] Qin Y., Ma Z., Dang X., Li W.E.I., Ma Q. (2014). Effect of resveratrol on proliferation and apoptosis of human pancreatic cancer MIA PaCa-2 cells may involve inhibition of the Hedgehog signaling pathway. Mol. Med. Rep..

[404] Ryu J., Yoon N.A., Seong H., Jeong J.Y., Kang S., Park N., Choi J., Lee D.H., Roh G.S., Kim H.J. (2015). Resveratrol induces glioma cell apoptosis through activation of tristetraprolin. Mol. Cells.

[405] Tian H., Yu Z. (2015). Resveratrol induces apoptosis of leukemia cell line K562 by modulation of sphingosine kinase-1 pathway. Int. J. Clin. Exp. Pathol..

[406] Tomic J., Mccaw L., Li Y., Hough M.R., Ben-david Y. (2013). Resveratrol has anti-leukemic activity associated with decreased *O*-GlcNAcylated proteins. Exp. Hematol..

[407] Vanamala J., Radhakrishnan S., Reddivari L., Bhat V.B., Ptitsyn A.B. (2011). Resveratrol suppresses human colon cancer cell proliferation and induces apoptosis via targeting the pentose phosphate and the talin-FAK signaling pathways—A proteomic approach. Proteome Sci..

[408] Varoni E.M., Faro A.F.L., Sharifi-Rad J., Iriti M. (2016). Anticancer molecular mechanisms of resveratrol. Front. Nutr..

[409] Carter L.G., D’Orazio J.A., Pearson K.J. (2014). Resveratrol and cancer: Focus on in vivo evidence. Endocr. Relat. Cancer.

[410] D’Incalci M., Steward W.P., Gescher A.J. (2005). Use of cancer chemopreventive phytochemicals as antineoplastic agents. Lancet Oncol..

[411] López-Lázaro M. (2008). Anticancer and carcinogenic properties of curcumin: Considerations for its clinical development as a cancer chemopreventive and chemotherapeutic agent. Mol. Nutr. Food Res..

[412] Burgos-Morón E., Calderón-Montaño J.M., Salvador J., Robles A., López-Lázaro M. (2010). The dark side of curcumin. Int. J. Cancer.

[413] Kurien B.T., Dillon S.P., Dorri Y., D’Souza A., Scofield R.H. (2011). Curcumin does not bind or intercalate into DNA and a note on the gray side of curcumin. Int. J. Cancer.

[414] Maru G.B. (2016). Understanding the molecular mechanisms of cancer prevention by dietary phytochemicals: From experimental models to clinical trials. World J. Biol. Chem..

[415] Zheng Y.Y., Viswanathan B., Kesarwani P., Mehrotra S. (2012). Dietary agents in cancer prevention: An immunological perspective. Photochem. Photobiol..

[416] Shukla Y., Singh R. (2011). Resveratrol and cellular mechanisms of cancer prevention. Ann. N. Y. Acad. Sci..

[417] Duvoix A., Blasius R., Delhalle S., Schnekenburger M., Morceau F., Henry E., Dicato M., Diederich M. (2005). Chemopreventive and therapeutic effects of curcumin. Cancer Lett..

[418] Nishino H., Tokuda H., Satomi Y., Masuda M., Osaka Y., Yogosawa S., Wada S., Mou X.Y., Takayasu J., Murakoshi M. (2004). Cancer prevention by antioxidants. BioFactors.

[419] Singh S., Khar A. (2006). Biological effects of curcumin and its role in cancer chemoprevention and therapy. Anticancer Agents Med. Chem..

[420] Bhat K.P.L., Pezzuto J.M. (2002). Cancer chemopreventive activity of resveratrol. Ann. N. Y. Acad. Sci..

[421] Aziz M.H., Kumar R.A.J., Ahmad N. (2003). Cancer chemoprevention by resveratrol: In vitro and in vivo studies and the underlying mechanisms (Review). Int. J. Oncol..

[422] Stepanic V., Gasparovic A.C., Troselj K.G., Amic D., Zarkovic N. (2016). Selected attributes of polyphenols in targeting oxidative stress in cancer. Curr. Top. Med. Chem..

[423] Mileo A.M., Miccadei S. (2016). Polyphenols as modulator of oxidative stress in cancer disease: New therapeutic strategies. Oxid. Med. Cell. Longev..

[424] Nafisi S., Adelzadeh M., Norouzi Z., Sarbolouki M.N. (2009). Curcumin binding to DNA and RNA. DNA Cell Biol..

[425] N’soukpoé-Kossi C.N., Bourassa P., Mandeville J.S., Bekale L., Tajmir-Riahi H.A. (2015). Structural modeling for DNA binding to antioxidants resveratrol, genistein and curcumin. J. Photochem. Photobiol. B Biol..

[426] Baharuddin P., Satar N., Fakiruddin K.S., Zakaria N., Lim M.N., Yusoff N.M., Zakaria Z., Yahaya B.H. (2016). Curcumin improves the efficacy of cisplatin by targeting cancer stem-like cells through p21 and cyclin D1-mediated tumour cell inhibition in non-small cell lung cancer cell lines. Oncol. Rep..

[427] Duarte V.M., Han E., Veena M.S., Salvado A., Jeffrey D., Liang L., Faull K.F., Srivatsan E.S., Wang M.B. (2010). Curcumin enhances the effect of cisplatin in suppression of head and neck squamous cell carcinoma via inhibition of IKKβ protein of the nuclear factor kB pathway. Mol. Cancer Ther..

[428] Chen J., Wang G., Wang L., Kang J., Wang J. (2010). Curcumin p38-dependently enhances the anticancer activity of valproic acid in human leukemia cells. Eur. J. Pharm. Sci..

[429] Epelbaum R., Schaffer M., Vizel B., Badmaev V., Bar-Sela G. (2010). Curcumin and gemcitabine in patients with advanced pancreatic cancer. Nutr. Cancer.

[430] Ferguson J.E., Orlando R.A. (2015). Curcumin reduces cytotoxicity of 5-Fluorouracil treatment in human breast cancer cells. J. Med. Food.

[431] Pandey A., Vishnoi K., Mahata S., Tripathi S.C., Misra S.P., Misra V., Mehrotra R., Dwivedi M., Bharti A.C. (2015). Berberine and curcumin target survivin and STAT-3 in gastric cancer cells and synergize actions of standard chemotherapeutic 5-fluorouracil. Nutr. Cancer.

[432] Yu Y., Kanwar S.S., Patel B.B., Nautiyal J., Sarkar F.H., Majumdar A.P. (2009). Elimination of colon cancer stem-like cells by the combination of curcumin and FOLFOX. Transl. Oncol..

[433] Gao J.-Z., Du J.-L., Wang Y.-L., Li J., Wei L.-X., Guo M.-Z. (2015). Synergistic effects of curcumin and bevacizumab on cell signaling pathways in hepatocellular carcinoma. Oncol. Lett..

[434] Guo Y., Li Y., Shan Q., He G., Lin J., Gong Y. (2015). Curcumin potentiates the anti-leukemia effects of imatinib by downregulation of the AKT/mTOR pathway and BCR/ABL gene expression in Ph+ acute lymphoblastic leukemia. Int. J. Biochem. Cell Biol..

[435] Hossain M.M., Banik N.L., Ray S.K. (2012). Synergistic anti-cancer mechanisms of curcumin and paclitaxel for growth inhibition of human brain tumor stem cells and LN18 and U138MG cells. Neurochem. Int..

[436] De Ruiz Porras V., Bystrup S., Martínez-Cardús A., Pluvinet R., Sumoy L., Howells L., James M.I., Iwuji C., Manzano J.L., Layos L. (2016). Curcumin mediates oxaliplatin-acquired resistance reversion in colorectal cancer cell lines through modulation of CXC-Chemokine/NF-κB signalling pathway. Sci. Rep..

[437] Zanotto-Filho A., Braganhol E., Klafke K., Figueiró F., Terra S.R., Paludo F.J., Morrone M., Bristot I.J., Battastini A.M., Forcelini C.M. (2015). Autophagy inhibition improves the efficacy of curcumin/temozolomide combination therapy in glioblastomas. Cancer Lett..

[438] Lee J.Y., Lee Y.M., Chang G.C., Yu S.L., Hsieh W.Y., Chen J.J.W., Chen H.W., Yang P.C. (2011). Curcumin induces EGFR degradation in lung adenocarcinoma and modulates p38 activation in intestine: The versatile adjuvant for gefitinib therapy. PLoS ONE.

[439] Björklund M., Roos J., Gogvadze V., Shoshan M. (2011). Resveratrol induces SIRT-1- and energy-stress-independent inhibition of tumor cell regrowth after low-dose platinum treatment. Cancer Chemother. Pharmacol..

[440] Osman A.M.M., Al-Malki H.S., Al-Harthi S.E., El-Hanafy A.A., Elashmaoui H.M., Elshal M.F. (2015). Modulatory role of resveratrol on cytotoxic activity of cisplatin, sensitization and modification of cisplatin resistance in colorectal cancer cells. Mol. Med. Rep..

[441] Buhrmann C., Shayan P., Kraehe P., Popper B., Goel A., Shakibaei M. (2015). Resveratrol induces chemosensitization to 5-fluorouracil through up-regulation of intercellular junctions, Epithelial-to-mesenchymal transition and apoptosis in colorectal cancer. Biochem. Pharmacol..

[442] Lee S.H., Koo B.S., Park S.Y., Kim Y.M. (2015). Anti-angiogenic effects of resveratrol in combination with 5-fluorouracil on B16 murine melanoma cells. Mol. Med. Rep..

[443] Díaz-Chavez J., Fonseca-Sanchez M.A., Arechaga-Ocampo E., Flores-Perez A., Palacios-Rodreguez Y., Domínguez-Góme G., Marchat L.A., Fuentes-Mera L., Mendoza-Hernandez G., Gariglio P. (2013). Proteomic profiling reveals that resveratrol inhibits HSP27 expression and sensitizes breast cancer cells to doxorubicin therapy. PLoS ONE.

[444] Kweon S.H., Song J.H., Kim T.S. (2010). Resveratrol-mediated reversal of doxorubicin resistance in acute myeloid leukemia cells via downregulation of MRP1 expression. Biochem. Biophys. Res. Commun..

[445] Casanova F., Quarti J., Ferraz Da Costa D.C., Ramos C.A., Da Silva J.L., Fialho E. (2012). Resveratrol chemosensitizes breast cancer cells to melphalan by cell cycle arrest. J. Cell. Biochem..

[446] Filippi-Chiela E.C., Thomé M.P., Bueno e Silva M.M., Pelegrini A.L., Ledur P.F., Garicochea B., Zamin L.L., Lenz G. (2013). Resveratrol abrogates the temozolomide-induced G2 arrest leading to mitotic catastrophe and reinforces the temozolomide-induced senescence in glioma cells. BMC Cancer.

[447] Harikumar K.B., Kunnumakkara A.B., Sethi G., Diagaradjane P., Anand P., Pandey M.K., Gelovani J., Krishnan S., Guha S., Aggarwal B.B. (2010). Resveratrol, a multitargeted agent, can enhance antitumor activity of gemcitabine in vitro and in orthotopic mouse model of human pancreatic cancer. Int. J. Cancer.

[448] Rigolio R., Miloso M., Nicolini G., Villa D., Scuteri A., Simone M., Tredici G. (2005). Resveratrol interference with the cell cycle protects human neuroblastoma SH-SY5Y cell from paclitaxel-induced apoptosis. Neurochem. Int..

[449] Shi X.P., Miao S., Wu Y., Zhang W., Zhang X.F., Ma H.Z., Xin H.L., Feng J., Wen A.D., Li Y. (2013). Resveratrol sensitizes tamoxifen in antiestrogen-resistant breast cancer cells with epithelial-mesenchymal transition features. Int. J. Mol. Sci..

[450] Singh N., Nigam M., Ranjan V., Zaidi D., Garg V.K., Sharma S., Chaturvedi R., Shankar R., Kumar S., Sharma R. (2011). Resveratrol as an adjunct therapy in cyclophosphamide-treated MCF-7 cells and breast tumor explants. Cancer Sci..

